# N^6^-methyladenosine RNA methyltransferase CpMTA1 mediates *CpAphA* mRNA stability through a YTHDF1-dependent m^6^A modification in the chestnut blight fungus

**DOI:** 10.1371/journal.ppat.1012476

**Published:** 2024-08-19

**Authors:** Lijiu Zhao, Xiangyu Wei, Fengyue Chen, Luying Yuan, Baoshan Chen, Ru Li

**Affiliations:** 1 State Key Laboratory for Conservation and Utilization of Subtropical Agro-bioresources, Guangxi Research Center for Microbial and Enzyme Engineering Technology, College of Life Science and Technology, Guangxi University, Nanning, China; 2 Guangxi Key Laboratory of Sugarcane Biology, College of Agriculture, Guangxi University, Nanning, China; Universidad Politécnica de Madrid: Universidad Politecnica de Madrid, SPAIN

## Abstract

In eukaryotic cells, N^6^-methyladenosine (m^6^A) is the most prevalent RNA epigenetic modification that plays crucial roles in multiple biological processes. Nevertheless, the functions and regulatory mechanisms of m^6^A in phytopathogenic fungi are poorly understood. Here, we showed that CpMTA1, an m^6^A methyltransferase in *Cryphonectria parasitica*, plays a crucial role in fungal phenotypic traits, virulence, and stress tolerance. Furthermore, the acid phosphatase gene *CpAphA* was implicated to be a target of CpMTA1 by integrated analysis of m^6^A-seq and RNA-seq, as *in vivo* RIP assay data confirmed that CpMTA1 directly interacts with *CpAphA* mRNA. Deletion of *CpMTA1* drastically lowered the m^6^A level of *CpAphA* and reduced its mRNA expression. Moreover, we found that an m^6^A reader protein CpYTHDF1 recognizes *CpAphA* mRNA and increases its stability. Typically, the levels of *CpAphA* mRNA and protein exhibited a positive correlation with CpMTA1 and CpYTHDF1. Importantly, site-specific mutagenesis demonstrated that the m^6^A sites, A^1306^ and A^1341^, of *CpAphA* mRNA are important for fungal phenotypic traits and virulence in *C*. *parasitica*. Together, our findings demonstrate the essential role of the m^6^A methyltransferase CpMTA1 in *C*. *parasitica*, thereby advancing our understanding of fungal gene regulation through m^6^A modification.

## Author summary

Numerous modifications have been found in eukaryotic mRNA, with m^6^A appearing to be the predominant modification. However, the biological roles and regulatory mechanisms of these m^6^A-modified enzymes are still largely unknown in filamentous fungi. Here, we characterized an m^6^A methyltransferase, CPMTA1, in *C*. *parasitica* using a combined analysis of multiple phenotypes and multi-omics. Based on our results, CpMTA1 plays a crucial role in fungal phenotypic traits, virulence, and stress tolerance. Furthermore, m^6^A-seq and RNA-seq identified the acid phosphatase gene *CpAphA* as a target of CpMTA1.The m^6^A methylation of *CpAphA* was verified by MeRIP-qPCR and MazF analysis. We also found that an m^6^A reader protein CpYTHDF1 could recognize *CpAphA* mRNA and increase its stability. Importantly, the m^6^A sites A^1306^ and A^1341^ of *CpAphA* mRNA are involved in the regulation of fungal phenotypic traits and virulence in *C*. *parasitica*. These results provide a comprehensive insight into the function and mechanism of CpMTA1-mediated m^6^A modification in fungi, as well as improve our understanding of m^6^A-related studies.

## Introduction

RNA modification is a post-transcriptional regulatory mechanism that influences RNA processing or metabolism [1]. N^6^-methyladenine (m^6^A) is the most common RNA methylation modification observed in diverse eukaryotic cells [2]. Three types of proteins dynamically and reversibly regulate m^6^A modification: m^6^A methyltransferases (“writers”), m^6^A demethylases (“erasers”), and m^6^A-binding proteins (“readers”) [3,4].

The m^6^A writer has been identified as a methyltransferase complex in many organisms, including mice, fruit flies, zebrafish, and humans. Typically, this complex consists of METTL3, METTL14, and additional protein subunits such as Zc3h13 or WTAP [5,6]. METTL3 is the primary m^6^A writer that assumes a pivotal catalytic function in methyl group transfer from the S-adenosylmethionine (SAM) to adenine [7]. Meanwhile, METTL14 facilitates substrate binding, while WTAP is necessary for RNA methylation and METTL3/14 localization [8]. Additionally, several other m^6^A methyltransferases have been identified, including METTL16, METTL5, ZCCHC4, and METTL4 [9]. METTL16 has been reported to methylate 3′ UTR of MAT2A mRNA thus regulating its mRNA expression [10]. METTL5 is an enzyme responsible for 18S rRNA m^6^A modification, while ZCCHC4 is involved in 28S rRNA modification [11]. The METTL4 protein acts as a methyltransferase in the regulation of pre-mRNA splicing by facilitating the m^6^A methylation of U2 snRNA [12]. Moreover, m^6^A erasers, such as AlkB homolog 5 (ALKBH5) and fat mass and obesity-associated protein (FTO), selectively remove the m^6^A modification from target mRNAs [13,14]. In addition, m^6^A recognizing proteins, also reported as readers, including the YTH homologous domain protein family, mediate mRNA degradation, splicing, and translation [15].

As a highly dynamic post-transcriptional regulation, m^6^A modification plays a significant role in various RNA metabolism processes, including mRNA structure, alternative splicing, processing, nuclear export, degradation, and translation [6]. For example, the exonic splicing of the adipogenic regulatory factor RUNX1T1 was regulated by FTO through modulating m^6^A levels of splice sites, thus influencing the process of differentiation [16]. YTHDC1, an m^6^A-binding protein, facilitates methylated mRNA transport from the nucleus to cytoplasm in HeLa cells [17]. YTHDF1 promotes RNA translation in the cytoplasm by interacting with initiation factors [18]. The mRNA degradation process is mediated by YTHDF2 through directly binding to m^6^A modification sites on LHPP and NKX3-1 [19].

Recent evidence suggests that m^6^A modification has been linked to multiple biological processes in various organism, including human circadian rhythms, stem cell differentiation, sex determination, viral replication, spermatogenesis, stress response, meiosis, obesity, cancer development and other diseases [6]. Moreover, an increasing number of studies have revealed essential roles for mRNA m^6^A modification in plant development, encompassing floral transition, trichome morphology, embryo development, shoot apical meristem proliferation, and fruit ripening [20]. The biological functions of m^6^A modification in fungi, however, have only been explored in a few species. In *Saccharomyces cerevisiae*, Ime4 (a homolog of mammalian methyltransferase METTL3) was identified as an essential component for m^6^A modification on yeast mRNA and regulates spore formation and meiosis, triglyceride metabolism, vacuolar morphology, mitochondrial morphological abnormalities, and dysfunction [21,22]. In the rice blast fungus *Magnaporthe oryzae*, the m^6^A writer MTA1, an orthologue of human METTL4, mediates m^6^A modification and regulates autophagy for fungal infection [5]. ALKB1 of *M*. *oryzae* was identified as an orthologue of m^6^A demethylase Alkbh1. YTH1 and YTH2 of *M*. *oryzae* were reported as orthologues of a human m^6^A reader protein. Knockout of these m^6^A-related genes in *M*. *oryzae* resulted in reduced virulence [23]. Two putative RNA m^6^A MTases (METTL3, METTL14) have been reported in *Rhizophagus irregularis* [24]. Recently, it was found that RNA modifications are associated with secondary metabolite biosynthesis of *Aspergillus flavus* [25]. Although these reports suggest that m^6^A modifications are important for fungi, the underlying mechanisms are largely unknown.

*Cryphonectria parasitica*, the causal agent of chestnut blight disease, almost destroyed American chestnut forests in the early 20th century [26]. Notably, some strains of the fungus contain a single-stranded, positive-sense RNA virus termed *Cryphonectria hypovirus* 1 (CHV1) that can serve as a biological control agent, as well as an important model to investigate virus-host interactions. After infection with CHV1, colonies of *C*. *parasitica* change from orange to white in colour, accompanied by a reduction in virulence, growth rate, sporulation, and female fertility [27,28]. The mechanisms of hypovirulence have been studied extensively through systemic analysis of transcriptome, proteome, metabolome, and DNA methylation, resulting in the identification of genes and pathways contributing to the phenotypes of interest [29–33]. Nevertheless, the regulatory mechanisms of reversible RNA methylation modification in *C*. *parasitica* have not yet been explored.

Our previous study found that CHV1 infection caused a significant decrease in the transcription level of *CpMTA1*, which encodes an m^6^A methyltransferase [33]. To understand the biological role of CpMTA1 in the chestnut blight fungus, we conducted a comprehensive analysis through the creation of *CpMTA1* deletion mutants. Furthermore, the target genes mediated by CpMTA1 were screened by the combined analysis of m^6^A-seq and RNA-seq results. Mechanistically, the m^6^A methylation of *C*. *parasitica* acid phosphatases (*CpAphA*) was further verified by MeRIP-qPCR and MazF analysis, and the interaction between *CpAphA* mRNA and CpYTHDF (the m^6^A reader, a homolog of human YTHDF1) was proven using RNA immunoprecipitation. Importantly, the functional significance of the m^6^A modification sites on *CpAphA* was examined by constructing a corresponding deletion mutant and site-specific mutagenesis. Overall, our findings provide novel insights into the roles and mechanisms of CpMTA1-mediated m^6^A modification in fungi.

## Results

### Characterization of m^6^A methyltransferase from *C*. *parasitica*

Among the observed decreased changes in transcription level caused by CHV1 infection, a gene tentatively identified as an m^6^A methyltransferase was chosen for further analysis [33]. The *CpMTA1* gene was characterized by exploration of the *C*. *parasitica* genome [28]. The coding region of the *CpMTA1* gene is composed of two exons with 336 amino acid residues and one intron. The CpMTA1 protein was found to contain a conserved MT-A70 domain (152–323 aa) through domain analysis, which was initially recognized as the SAM-binding subunit of the human N^6^-adenosine-methyltransferase (MTase) and exhibits specific methylation activity towards adenines in pre-mRNAs [5]. A phylogenetic analysis was performed to further investigate the relationship between the CpMTA1 protein and its homologous sequences. The result showed that CpMTA1 is evolutionarily conserved in filamentous fungi, suggesting similar importance for other fungi (S1 Fig).

**Fig 1:**
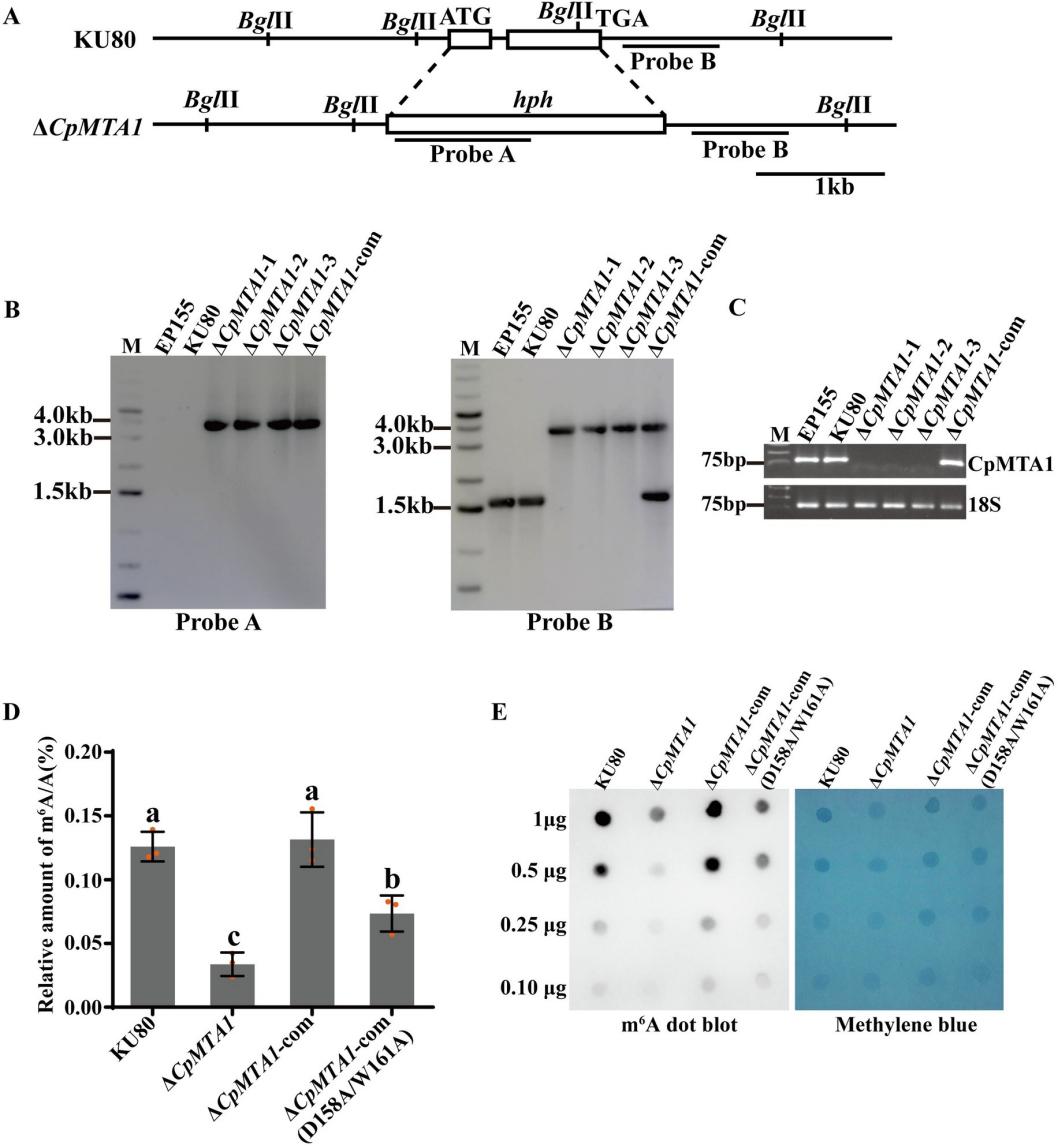
Deletion of *CpMTA1* affects the m^6^A RNA methylation of *Cryphonectria parasitica*. (A): Schematic diagram of the *CpMTA1* gene deletion strategy. Fragment on the *hph* gene (probe A) and fragment on the right arm (probe B) were used to distinguish the fragment size of the wild-type strain and Δ*CpMTA1* mutants in Southern blot. Scale bar = 1 kb. (B): The Southern blot analysis was conducted in Δ*CpMTA1* mutants using probe A (left) and probe B (right). Fungal total DNAs were digested with *Bgl* II and separated in the agarose gel by electrophoresis, then blotted using probe A and probe B, respectively. (C): The *CpMTA1* gene deletion was confirmed at the RNA level by RT-PCR. As an internal reference gene, 18S rRNA was used. (D): The relative amount of m^6^A/A in *CpMTA1* mutant strains was calculated with RNA methylation quantitative kit. There are significant differences between samples indicated by different letters on the bars. (ANOVA followed by Tukey’s test, p < 0.05). (E): m^6^A dot blot analysis of m^6^A levels using a specific m^6^A antibody. RNA from different samples was extracted, spotted onto membranes, and incubated with an m^6^A antibody before being detected using the ECL system. Methylene blue staining served as a loading control.

To investigate the biological function of the *CpMTA1* in *C*. *parasitica*, we constructed the *CpMTA1* knockout mutant using replacement with a hygromycin resistance gene (*hph*). The single-spored Δ*CpMTA1* mutants were screened by PCR, then confirmed by Southern blot and RT-PCR (Fig 1A–1C). To confirm the observed phenotypes of the mutants were caused by the deletion of *CpMTA1*, the Δ*CpMTA1* mutants were complemented by re-introducing a copy of the wild-type *CpMTA1* gene (S2 Fig).

It has been reported that METTL3 possesses a catalytic DPPW motif that enables its selective recognition of m^6^A [34]. According to active site prediction and amino acid sequence alignment, a conserved DPPW (D158-W161) motif was also identified on CpMTA1, suggesting that this may be necessary for the methyltransferase activity (S3 Fig). Consequently, we introduced mutations to convert the m^6^A recognition sites from DPPW to APPA. To further illustrate the structural basis for the mutation of CpMTA1, we employed a structure prediction software, AlphaFold2, to construct a 3D model structure of CpMTA1 and APPA mutant proteins using their respective amino acid sequences. We reanalyzed the structure of active sites in CpMTA1 and observed that substitutions of Asp158 and Trp161 to Ala would likely affect the formation of hydrogen bonds with surrounding residues and possibly influence the substrate recognition of the enzyme (S4 and S5 Figs). The genes with confirmed sequencing of CpMTA1 mutations were incorporated into the *CpMTA1* deletion mutant to construct the catalytic inactive mutant Δ*CpMTA1*-com (D158A/W161A).

To determine whether CpMTA1 is responsible for RNA m^6^A methylation in *C*. *parasitica*, we compared the total m^6^A RNA methylation levels of the parent strain KU80, Δ*CpMTA1*, Δ*CpMTA*-com and Δ*CpMTA1*-com (D158A/W161A) (Fig 1D). The amount of m^6^A RNA in the Δ*CpMTA1* mutant was 0.034 ± 0.009% of the total RNA, which was 26.98% of that in the parent strain KU80 (0.126 ± 0.011% of the total RNA), showing a significant reduction of m^6^A RNA modification. Compared with KU80, the m^6^A modification level was fully restored in the complemented strain Δ*CpMTA*-com, but not the catalytically inactive mutant (D158A/W161A). Similar results were obtained when an m^6^A dot blot assay was used. As shown in Fig 1E, the m^6^A RNA methylation level of Δ*CpMTA1* was lower than that of KU80 strain at all detected RNA amounts. These results demonstrate that CpMTA1 appears to be involved in mRNA m^6^A methylation of *C*. *parasitica*.

### *CpMTA1* is necessary for fungal phenotypic traits, virulence, and stress tolerance

**Fig 2:**
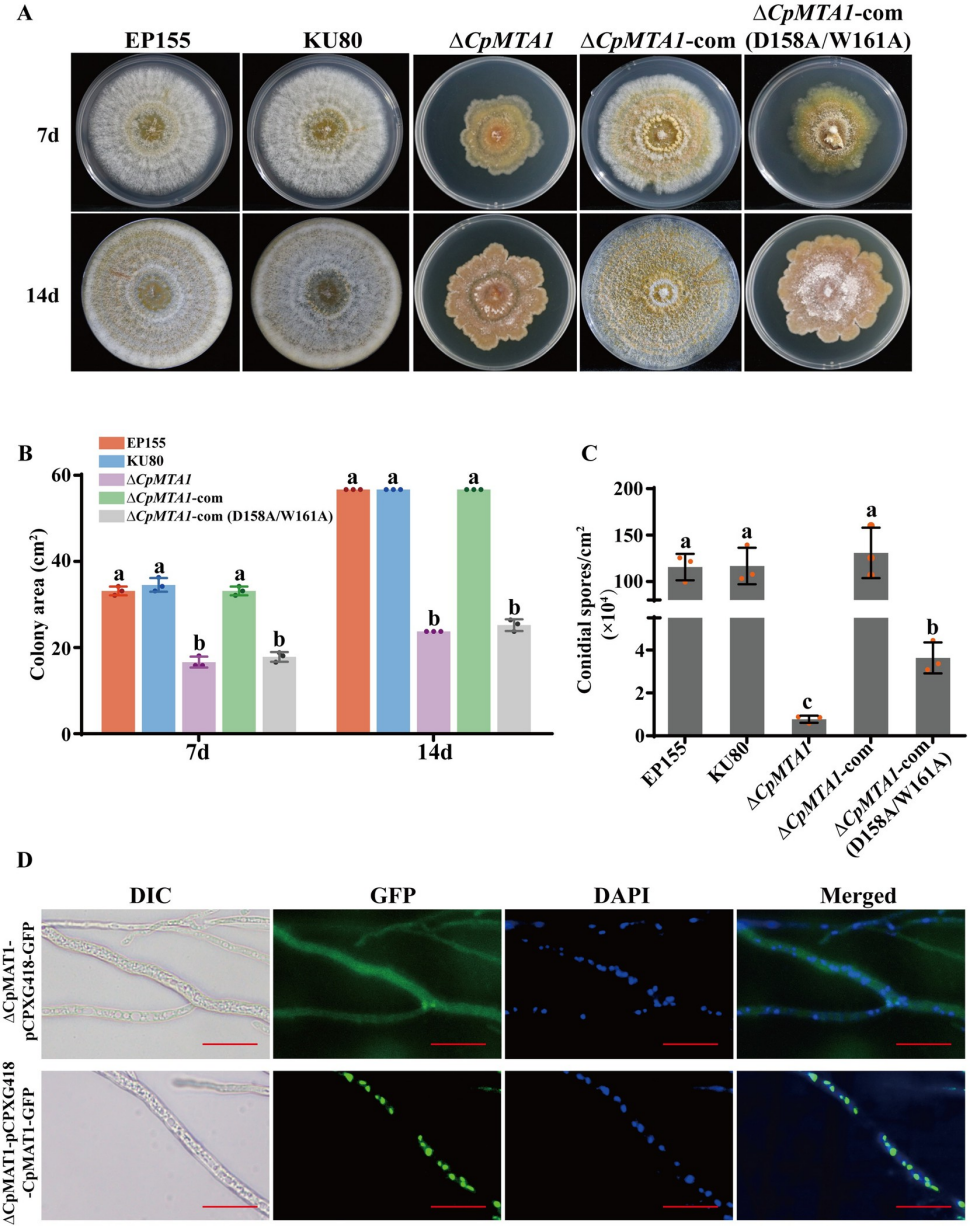
Phenotype analysis of *CpMTA1* mutant strains. (A): Colony morphology of the mutants on PDA medium. Photographs were taken at 7 and 14 days after inoculation. The wild-type EP155, parental strain KU80, *CpMTA1* deletion strain Δ*CpMTA1*, complementary strain Δ*CpMTA1*-com, and mutant complementation strain Δ*CpMTA1*-com (D158A/W161A) were shown. (B): Mutant colony area were measured at day 7 and 14 post-inoculation. (C): Sporulation levels of the tested strains. Spores were calculated on day 14. There are significant differences between samples indicated by different letters on the bars (ANOVA followed by Tukey’s test, p < 0.05). (D): Subcellular localization of CpMTA1-GFP fusion protein. Images were taken using light microscopy and fluorescence microscopy, respectively. DAPI staining was performed to visualize the nuclei. Scale bar, 20 μm.

To study the roles of *CpMTA1* in the mycelial growth and sporulation of *C*. *parasitica*, the mutants and WT strains were compared on PDA plates. The Δ*CpMTA1* mutant exhibited a growth rate defect and displayed an irregular colony margin compared to the WT strain EP155 and parent strain KU80 (Fig 2A and 2B). Moreover, the deletion of *CpMTA1* led to a significant decrease in sporulation (Fig 2C). These abnormal phenotypes could be successfully rescued by the complementation of the WT-*CpMTA1*, but not the catalytically inactive mutant (D158A/W161A). Additionally, we found that CpMTA1 is located in the nucleus, which is consistent with a previous report (Fig 2D) [35].

**Fig 3:**
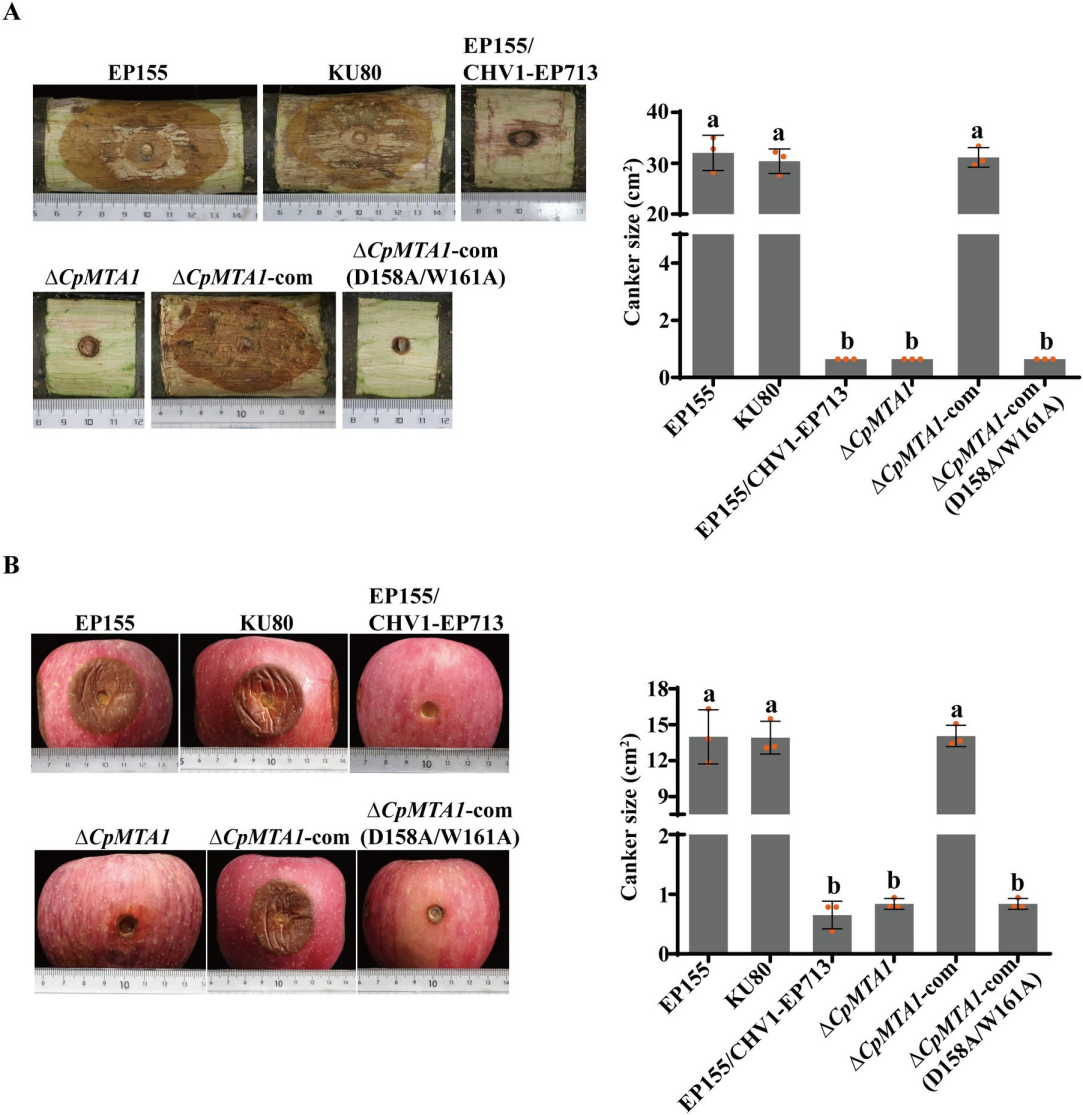
Virulence assay of *CpMTA1* mutant strains. (A): Cankers were induced using the indicated strains on dormant stems of Chinese chestnut. The stems were inoculated and then maintained at 26°C, and the cankers were subsequently measured and photographed at 25 days post-inoculation. (B): Cankers were induced using the tested strains on Red Fuji apples. The apples were inoculated and kept at 26°C, and cankers were measured and photographed at 10 days post-inoculation. There are significant differences between samples indicated by different letters on the bars (ANOVA followed by Tukey’s test, p<0.05).

To further determine whether CpMTA1 is involved in fungal virulence, the virulence test was conducted on chestnut stems and apples. As shown in Fig 3, the WT strain EP155 and KU80 were highly virulent, while the hypovirus-infected strain EP155/CHV1-EP713 incited very small cankers. The result also showed a significant virulence reduction of the Δ*CpMTA1* mutants. The Δ*CpMTA1*-com strain restored the virulence to resemble that of wild-type strains, but the site-specific mutant exhibited similar virulence to Δ*CpMTA1*.

Furthermore, using oxidative stress (H_2_O_2_), osmotic stress (NaCl), or cell wall integrity stress (Congo red or SDS), the Δ*CpMTA1* mutants were examined for stress tolerance. The Δ*CpMTA1* strains displayed more sensitivity to both H_2_O_2_, SDS, and NaCl compared to EP155 and KU80 strains (S6 Fig), whereas no significant difference was found on Congo red. Besides, the hypovirus CHV1-EP713 was introduced into Δ*CpMTA1* strains through anastomosis with EP155/CHV1-EP713 to investigate the effect of *CpMTA1* deletion on hypovirus infection. Hypovirus-infected Δ*CpMTA1* strains exhibited a decreased growth rate and reduced pigmentation. Analysis of viral dsRNA levels revealed comparable accumulation of CHV1-EP713 in both Δ*CpMTA1*/CHV1-EP713 and EP155/CHV1-EP713 (S7 Fig). Collectively, these results support that CpMTA1 plays a crucial role in fungal phenotypic traits, virulence, and stress tolerance. Moreover, the conserved active sites (D158/W161) are important in regulating CpMTA1 function *in vivo*.

### Identification of m^6^A modification in *C*. *parasitica* through transcriptome-wide m^6^A-seq and RNA-seq assays

**Fig 4:**
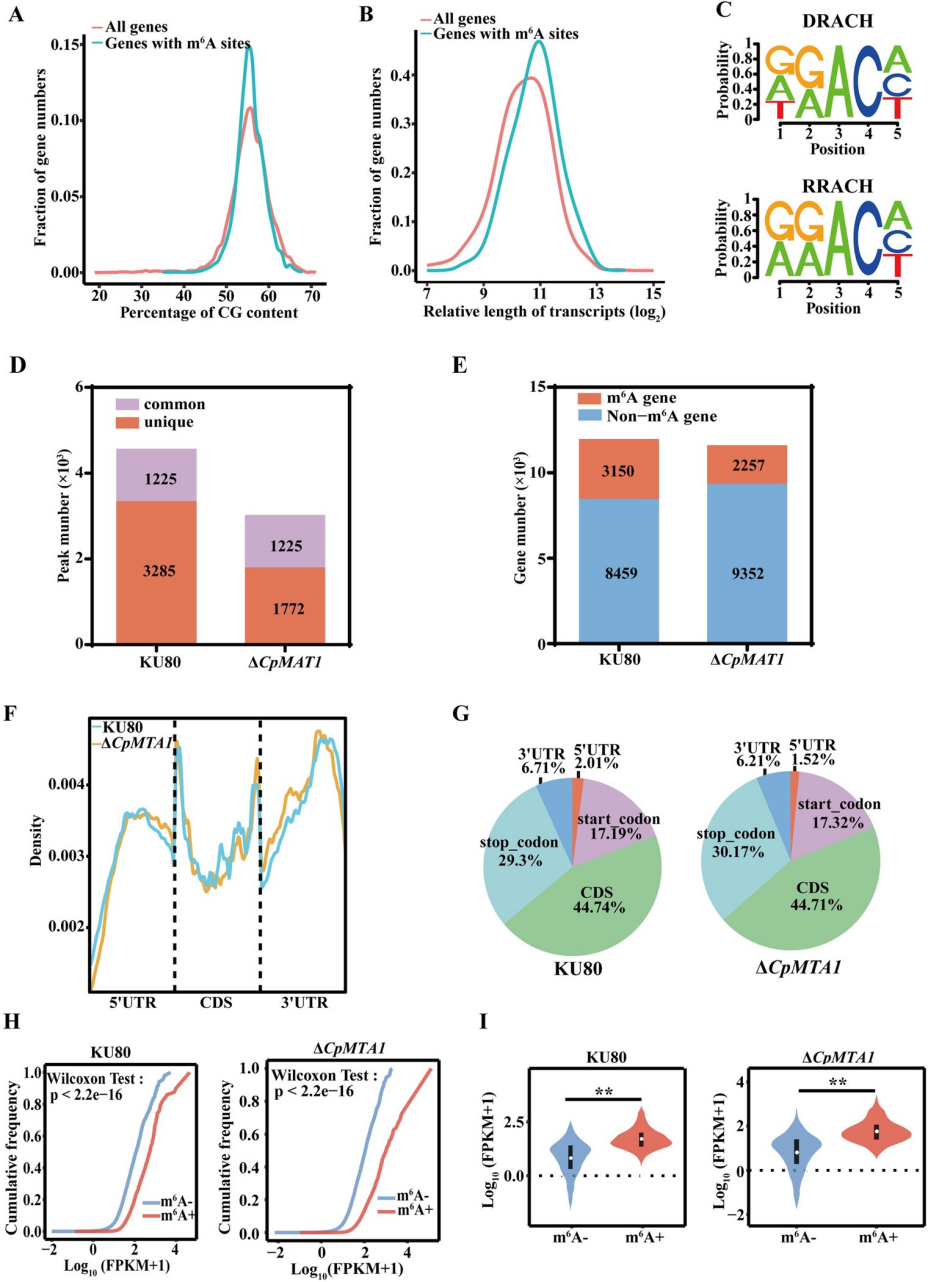
Characterization of m^6^A modification in the KU80 and Δ*CpMTA1* mutant. (A): Line plots displaying the percentage of CG content of all genes harboring m^6^A modifications identified through m^6^A-seq. (B): Line plots displaying the relative length of transcripts in all genes harboring m^6^A modifications. (C): Predominant consensus motif found using HOMER with m^6^A-seq peaks in KU80. Number of m^6^A peaks (D) and m^6^A-modified genes (E) identified by m^6^A-seq in KU80 and Δ*CpMTA1*. (F): Distribution of m^6^A peaks along the whole mRNA transcripts divided into the 5’UTR, CDS, and 3’UTR. (G): The proportions of m^6^A peaks distributed in the indicated regions in KU80 and Δ*CpMTA1*. (H): In KU80 and the Δ*CpMTA1* mutant, genes are divided into two categories according to the number of m^6^A sites in each gene. m^6^A- gene (m^6^A site = 0), m^6^A+ gene (m^6^A site > = 1). (I): Cumulative distribution of mRNA expression changes between m^6^A-modified genes (m^6^A+) and non-modified genes (m^6^A-). **, p<0.01.

To reveal the mechanism of CpMTA1 in m^6^A modification of *C*. *parasitica*, we conducted transcriptome-wide m^6^A sequencing (m^6^A-seq) and RNA sequencing (RNA-seq) analysis using the parent strain KU80 and Δ*CpMTA1* mutant. We compared the m^6^A peaks detected between m^6^A immunoprecipitated (IP) samples and the corresponding input samples as control. Firstly, we analyzed the m^6^A peaks identified in KU80 to determine the distribution of peaks across the genome in *C*. *parasitica*. m^6^A methylation shows a preference for genes with 50–60% CG content (Fig 4A) and longer transcript lengths (Fig 4B), compared to the overall genome of *C*. *parasitica*. Furthermore, motif analysis of enriched m^6^A peaks revealed that DRACH and RRACH (D = A, G, U; R = A, G; H = A, U, C) were the most frequently conserved consensus motifs (Fig 4C).

In all, m^6^A-seq found 4510 and 2997 m^6^A peaks from 3150 and 2257 m^6^A-modified genes in KU80 and Δ*CpMTA1* samples, respectively (Fig 4D and 4E and S1 Table). Compared to KU80, 3285 peaks disappeared in Δ*CpMTA1*, and 1772 peaks appeared. The other 1225 peaks were found in both KU80 and Δ*CpMTA1* strains. The *CpMTA1* deletion mutant exhibits a noticeable decrease in the number of m^6^A peaks. Since CpMTA1 is an m^6^A methyltransferase, the 3285 KU80-unique peaks are expected to contain potential targets of CPMTA1. We then performed gene set enrichment analysis (GSEA) to investigate the potential downstream pathways regulated by CpMTA1. As shown in S8 Fig and S2 Table, CpMTA1 was positively correlated with metabolic pathways, amino acid biosynthesis, and oxidative phosphorylation. In contrast, a negative correlation was found between CpMTA1 and MAPK signalling pathway, meiosis, and cell cycle.

Furthermore, we investigated the m^6^A distribution patterns within KU80 and Δ*CpMTA1*. After segment normalization by the total length of each gene portion, the results showed that m^6^A peaks were mainly distributed in the coding sequences (CDS) and the 3′ terminate (near the stop codon) (Figs 4F, 4G and S9A and S3 Table). There was no notable difference in m^6^A peak distribution between the two strains. To further examine whether gene expression is related to m^6^A modification, the expression abundance with or without m^6^A modification was compared. We found that the expression levels of m^6^A-modified genes were significantly higher than those of non-m^6^A-modified genes in two strains (Fig 4H and 4I).

### Combined analysis of m^6^A-seq and RNA-seq data of KU80 and Δ*CpMTA1* samples

**Fig 5:**
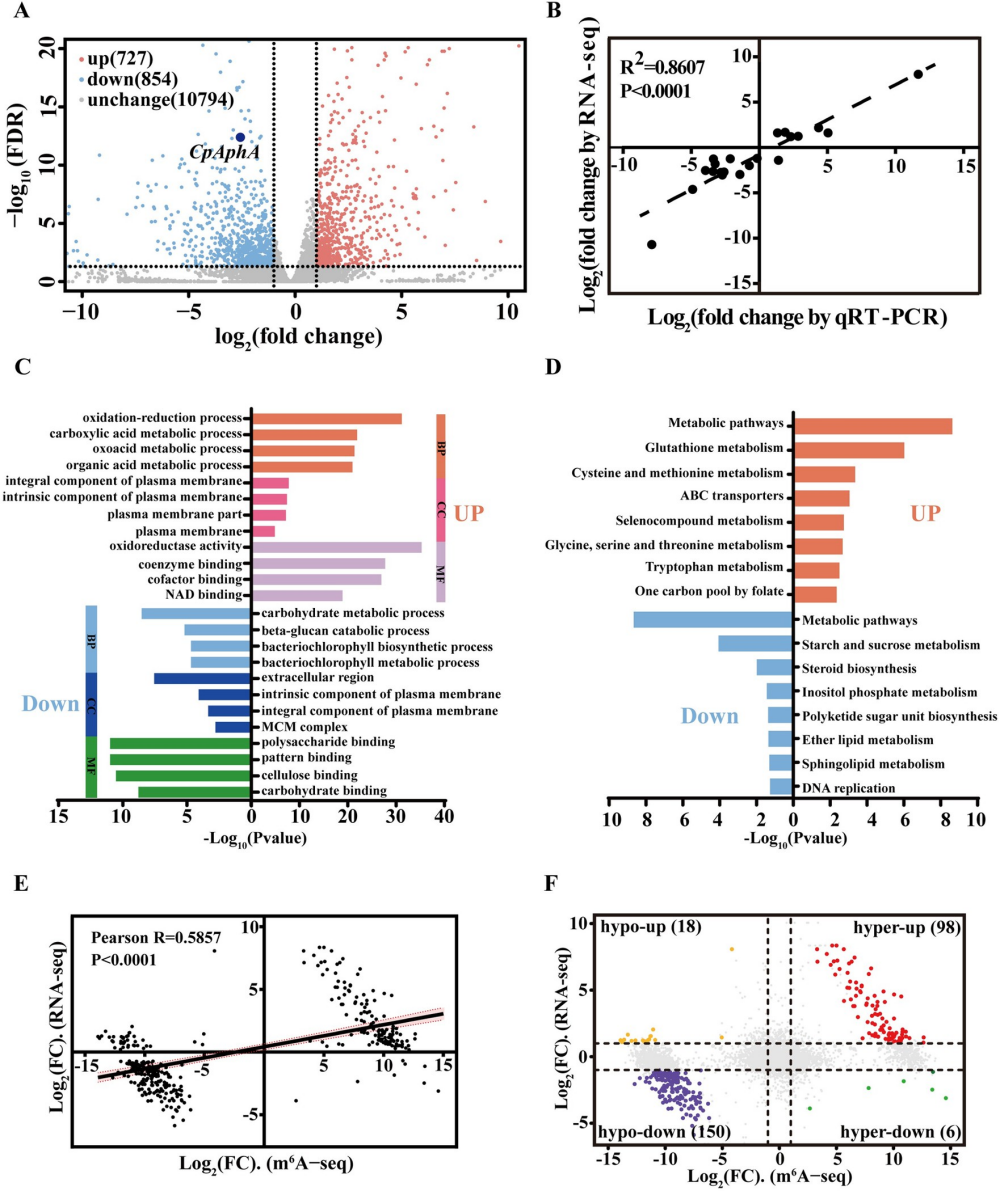
Combined analysis of m^6^A-seq and RNA-seq data of the KU80 and Δ*CpMTA1* samples. (A): RNA-seq data from KU80 and Δ*CpMTA1*. The cutoff criteria for differentially expressed genes was log_2_|FC|≥1 and FDR≤0.05 (red color indicates an increase and blue color represents a decrease in mRNA expression.) (B): Confirmation of the expression of 21 randomly selected DEGs by qRT-PCR analysis. Log_2_FC was calculated from three samples. The coefficient of determination (R^2^) was displayed. (C): GO-based enrichment analysis of DEGs in terms of biological process (BP), cell component (CC) and molecular function (MF). (D): KEGG pathway enrichment analysis of DEGs. (E): A dot plot showing the log_2_ (FC) of mRNA expression in relation to the log_2_ (FC) of differential m^6^A level. (F): A nine-image map to display the distribution of genes with a significant change in both m^6^A level and mRNA expression level in Δ*CpMTA1* compared to the control KU80. Yellow represents decreased m^6^A level and increased mRNA (hypo-up). Red represents increased m^6^A level and mRNA (hyper-up). Purple represents decreased m^6^A level and mRNA (hypo-down). Green represents increased m^6^A level and decreased mRNA (hyper-down).

To investigate the impact of m^6^A on gene expression at the transcriptional level, we compared the abundance between KU80 and Δ*CpMTA1* samples by performing differentially expressed gene (DEG) analysis. A total of 727 and 854 genes showed a significant increase and decrease, respectively (Fig 5A and S4 Table). To verify the RNA-seq results, a total of 21 DEGs were selected for qRT-PCR analysis. The Pearson correlation analysis between qRT-PCR and RNA-seq revealed that the values were consistent (correlation coefficient R^2^ = 0.8607, p < 0.0001), confirming the reliability of the RNA-seq (Fig 5B). Furthermore, these DEGs were used to perform Gene Ontology (GO) enrichment analysis and Kyoto Encyclopedia of Genes and Genomes (KEGG) pathway analysis (Fig 5C and 5D). In detail, the DEGs downregulated in Δ*CpMTA1* compared to those in KU80 were significantly enriched in carbohydrate metabolic process, polysaccharide binding (GO), metabolic pathways, starch and sucrose metabolism, and inositol phosphate metabolism (KEGG). Compared to those in KU80, the upregulated genes were mostly involved in oxidation-reduction process, oxidoreductase activity (GO), glutathione metabolism, cysteine and methionine metabolism (KEGG).

Subsequently, the correlation between differentially methylated genes and their corresponding mRNA expression levels was assessed by combining m^6^A-seq and RNA-seq data. It was found that the m^6^A methylation level exhibited a significantly positive association with gene expression levels (Fig 5E). A total of 272 differentially methylated and differentially expressed genes (log_2_|FC|≥1 and FDR≤0.05) were shown in the nine-quadrant diagram. Most of these genes were hypomethylated and downregulated (hypo-down, 150/272), and hypermethylated and upregulated (hyper-up, 98/272). In contrast, 18 genes were hypomethylated and upregulated (hypo-up). Only six genes showed hypermethylated and down-expression (hyper-down) (Fig 5F and S5 Table).

### *CpAphA* is a direct target gene of CpMTA1

**Fig 6:**
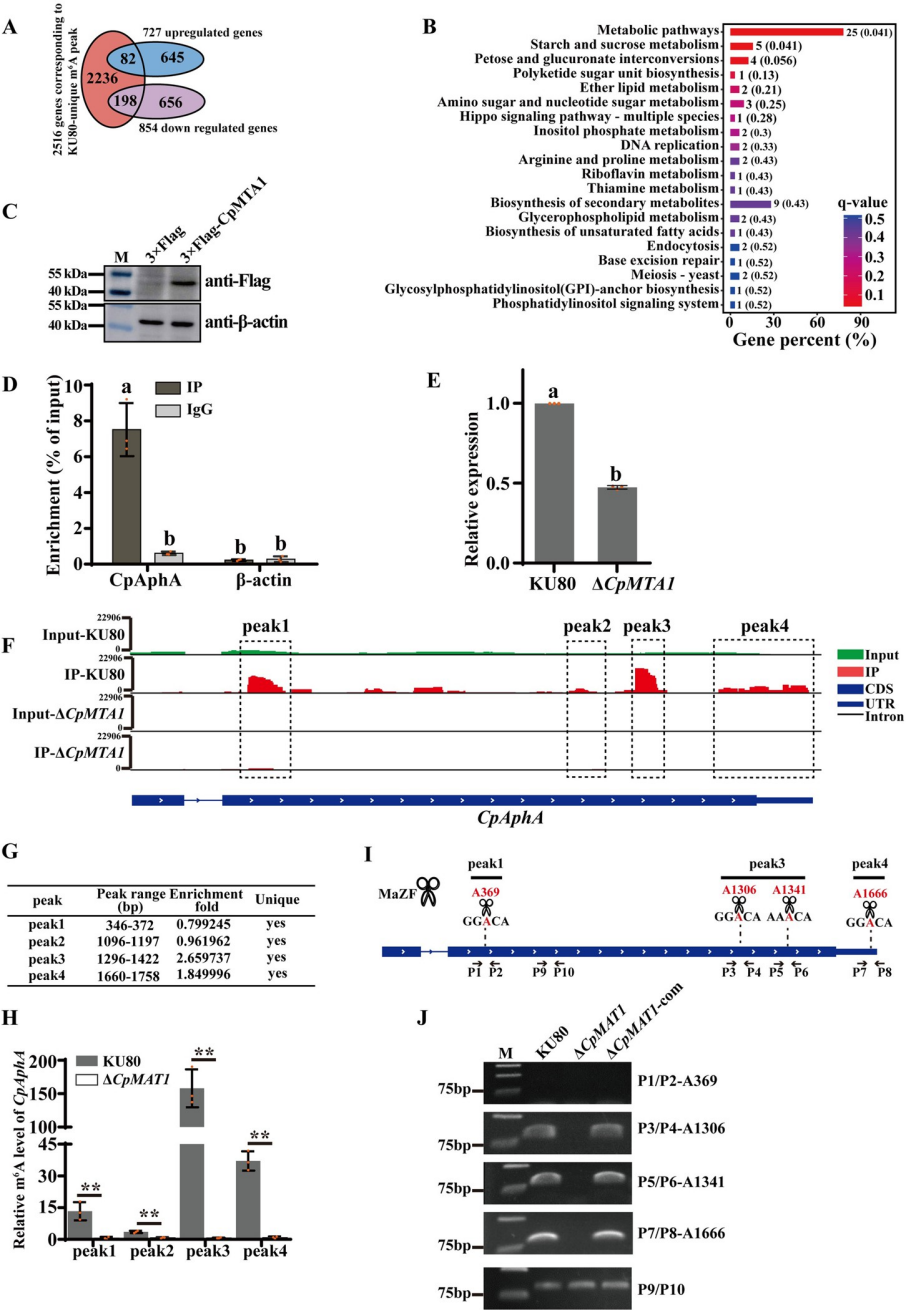
*CpAphA* is a direct target gene of CpMTA1. (A): Venn diagram showing the numbers of overlapped genes between 2516 genes corresponding to KU80-unique peaks and RNA-seq data. (B): Enrichment of KEGG metabolic pathways in 198 genes with KU80-unique m^6^A peak and downregulated expression. (C): Verification of EP155/3×Flag-CpMTA1 strain by western blotting with an anti-flag antibody or anti-β-actin antibody (control). (D): RIP assay confirmed the association between *CpAphA* and CpMTA1. RIP assay was performed using anti-flag antibody in EP155/3×flag-CpMTA1 strain. β-actin was used as a negative control. The fold enrichment values were normalized to that of Input. (E): qRT-PCR verification of *CpAphA* expression in KU80 and Δ*CpMTA1*. There are significant differences between samples indicated by different letters on the bars (ANOVA followed by Tukey’s test, p<0.05). (F): Integrative genomics viewer (IGV) plots displaying m^6^A peaks in *CpAphA* transcript. Y-axis indicates normalized numbers of reads count. The black rectangles indicate that m^6^A peaks with significantly decreased m^6^A enrichment in Δ*CpMTA1* compared to KU80. (G): Fold enrichment of IP/input for peaks in the window. (H): MeRIP-qPCR analysis of m^6^A levels of *CpAphA*. The mRNA of the KU80 and Δ*CpMTA1* mutant were used for m^6^A-IP assay with an anti-m^6^A antibody. Subsequent MeRIP-qPCR was conducted using IP assay products as the template. The asterisk represents a statistically significant difference from KU80 (p<0.01). (I): Schematic diagram of MazF enzyme assay. Scissors represent endonuclease MazF which preferentially cleaves RNA at ACA sites but not at m^6^ACA sites. The MazF ACA site within the methylation site was highlighted in red. (J): PCR amplification of cDNA prepared from MazF-digested mRNA from KU80, Δ*CpMTA1* and Δ*CpMTA1-*com. P1/P2, P3/P4, P5/P6 and P7/P8 primer locations relative to the probed methylation site were shown in (I). The control primers P9/P10, which do not flank an ACA site, were used as negative control.

According to the result shown in Fig 4D, we identified 3285 KU80-unique m^6^A peaks covering 2516 genes. Overlap analysis of 2516 genes and RNA-seq data revealed 198 downregulated genes with lost m^6^A modification in Δ*CpMTA1* mutant are expected to contain genuine targets of CpMTA1 (Fig 6A and S6 Table). KEGG pathway enrichment analysis of 198 downregulated genes suggested that many metabolic pathways were associated with m^6^A methylation, which involves starch and sucrose metabolism, pentose and glucuronate interconversions, ether lipid metabolism, and inositol phosphate metabolism (Fig 6B and S7 Table).

Among these 198 genes, 80 have multiple m^6^A peaks (S9B Fig and S8 Table). Ries *et al*. [36] has demonstrated that m^6^A regulates the fate of cytosolic mRNA by interacting with m^6^A-binding protein. This interaction is particularly effective for polymethylated mRNAs, which can bind multiple m^6^A-binding proteins with high affinity. In contrast, singly methylated mRNA has low binding affinity and cannot mediate such functions effectively. Based on this information, the top six polymethylated mRNAs from S8 Table were selected for further RNA Immunoprecipitation (RIP) assay to investigate their potential targeting by CpMTA1. The results showed a substantial enrichment of *CpAphA* transcripts, but not the other five genes, indicating a direct binding of CpMTA1 to *CpAphA* mRNA (Figs 6C, 6D and S10). *CpAphA* was annotated to acid phosphatase (XP_040780407.1) in NCBI (S11 Fig). Therefore, it is suggested that *CpAphA* may be a target gene of CpMTA1. Subsequent qRT-PCR assays showed a significant decrease in the expression level of *CpAphA* mRNA upon deletion of *CpMTA1* (Fig 6E), consistent with the RNA-seq results. Moreover, as shown in Fig 6F and 6G, *CpAphA* mRNA derived from KU80, but not Δ*CpMTA1*, harbors four m^6^A peaks in its CDS and 3′ UTR. We then performed methylated RNA Immunoprecipitation (MeRIP) followed by *CpAphA-*specific qPCR to verify the change of *CpAphA* m^6^A modification. As expected, the four *CpAphA* m^6^A peaks exhibited strongly decreased methylated levels in Δ*CpMTA1*, confirming that CpMTA1 is the responsible methyltransferase that methylates the CDS and 3′ UTR of *CpAphA* (Fig 6H).

To further identify exact methylation sites of four m^6^A peaks in *CpAphA* transcripts, a methylation-sensitive RNA restriction enzyme MazF was used, which cleaves RNA at ACA sites but not m^6^ACA sites [37]. The ACA sequence is part of the DRACA motif observed in the *CpAphA* m^6^A peak 1, 3, and 4 (Fig 6I). Through motif screening analysis, we speculated that A396, A1306, A1341, and A1666 may be key methylation modification sites, which were located in *CpAphA* m^6^A peak 1, 3, and 4. The purified mRNA from KU80, Δ*CpMTA1*, and Δ*CpMTA1*-com were digested with MazF and then reverse transcribed to synthesize cDNA, respectively. PCR amplification of the KU80 and Δ*CpMTA1*-com cDNA using primers P3/P4, P5/P6 and P7/P8 yielded a corresponding product, while no product was obtained from Δ*CpMTA1* cDNA. Meanwhile, no product was obtained from both tested strains using P1/P2 primers for amplification. PCR with P9/P10 primers was used as negative control (Fig 6J). Thus, the A1306, A1341, A1666 are protected from MazF cleavage in mRNA prepared from KU80 and Δ*CpMTA1*-com. This result supports that *CpAphA* is methylated at the A1306, A1341, and A1666 positions, which are regulated by the methyltransferase CpMTA1.

### CpMTA1 mediates the *CpAphA* mRNA stability through a CpYTHDF1-dependent m^6^A modification

**Fig 7:**
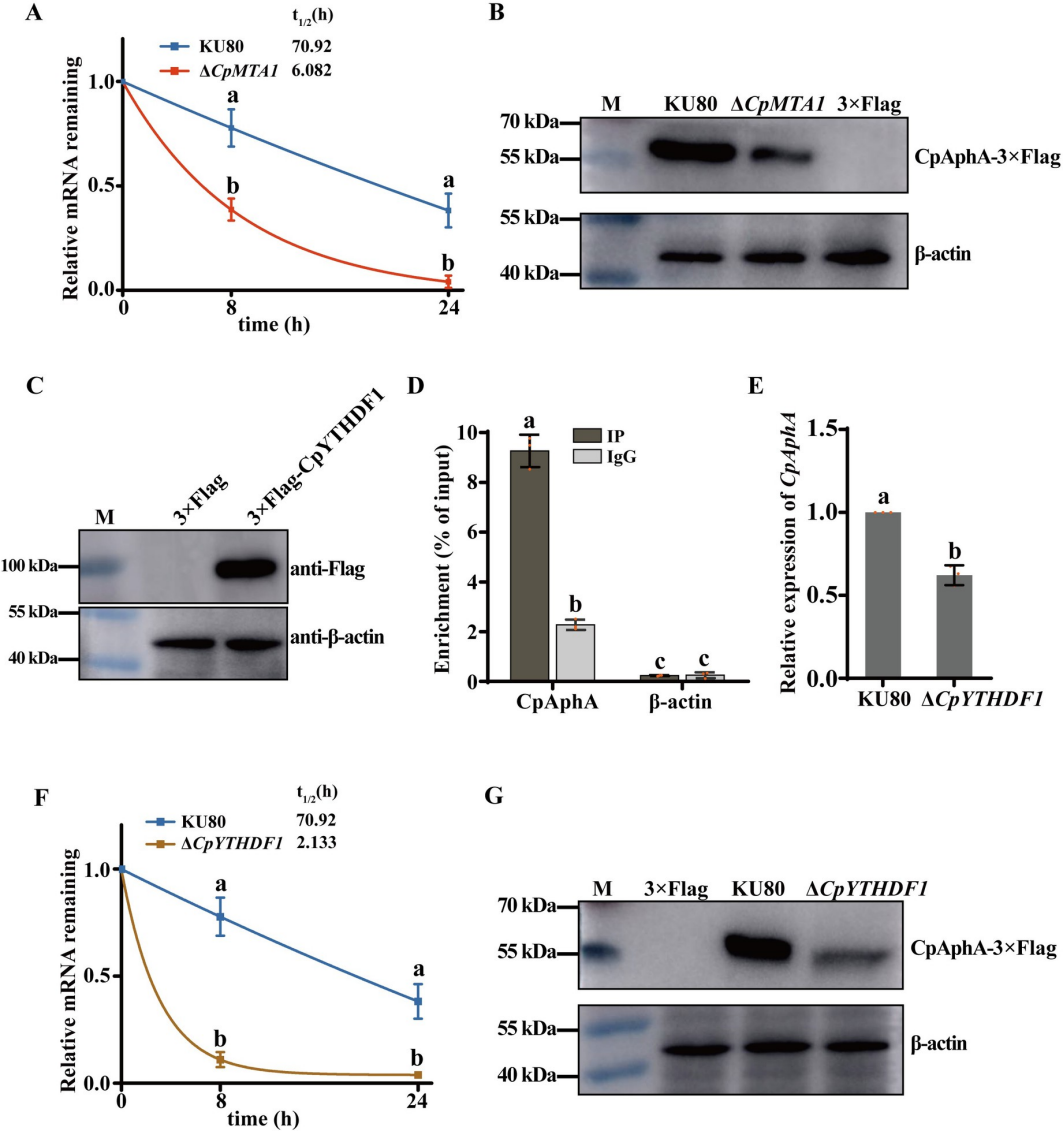
The stability of *CpAphA* mRNA is regulated by CpMTA1 via a CpYTHDF1-dependent m^6^A modification. (A): The expression levels of *CpAphA* mRNA in KU80 and Δ*CpMTA1* were analyzed by qRT-PCR at indicated time points after actinomycin D treatment. The mRNA half-life (t_1/2_) of *CpAphA* was evaluated with a nonlinear regression model. (B): The protein expression of CpAphA in KU80 and Δ*CpMTA1* were determined by western blotting with an anti-flag antibody or anti-β-actin antibody (control). (C): Verification of EP155/3×Flag-CpYTHDF1 strain by western blotting with an anti-flag antibody or anti-β-actin antibody (control). (D): RIP assay confirmed the association between *CpAphA* and m^6^A reader protein CpYTHDF1. RIP assay was performed using the anti-flag antibody in EP155/3×flag-CpYTHDF1 strain. The negative control was β-actin. The fold enrichment values were normalized to that of Input. (E): qRT-PCR analysis of *CpAphA* mRNA expression in KU80 and Δ*CpYTHDF1*. (F): The expression levels of *CpAphA* mRNA in KU80 and Δ*CpYTHDF1* were determined by qRT-PCR at indicated time points after actinomycin D treatment and the decay rate of *CpAphA* was evaluated with a nonlinear regression model. (G): The protein expression of CpAphA in KU80 and Δ*CpYTHDF1* was determined by western blotting with an anti-flag antibody or anti-β-actin antibody (control). There are significant differences between samples indicated by different letters on the bars (ANOVA followed by Tukey’s test, p < 0.05).

Previous reports suggest that RNA m^6^A modifications affect mRNA stability [38,39], which may explain why *CpAphA* mRNA expression is reduced in Δ*CpMTA1*. Therefore, we assessed the *CpAphA* mRNA decay rate in KU80 and Δ*CpMTA1* using the transcription inhibitor actinomycin D. As presented in Fig 7A, knockout of *CpMTA1* significantly shortened the half-life of *CpAphA* mRNA, suggesting that *CpMTA1*-induced repression of *CpAphA* mRNA expression is at least in part due to the decreased stability of *CpAphA* mRNA. Furthermore, western blot assay revealed that the protein level of CpAphA was significantly decreased in Δ*CpMTA1* (Fig 7B).

Meanwhile, m^6^A “readers” play an essential role in controlling the fate of the m^6^A marked mRNA [40]. To identify the specific m^6^A reader of *CpAphA*, we searched for the homologous protein in the *C*. *parasitica* genome database using human m^6^A reader YTHDF1/2/3 as a query, respectively. As a result, we identified a homologous protein of YTHDF1, but not for YTHDF2/3. We named the *C*. *parasitica* m^6^A reader protein (XP_040773278) as CpYTHDF1, which contains a YTH domain. Furthermore, RIP assays confirmed the direct interaction between the CpYTHDF1 and *CpAphA* mRNA in *C*. *parasitica* (Fig 7C and 7D). Additionally, *CpAphA* mRNA level was significantly decreased upon the deletion of *CpYTHDF1*, indicating that *CpYTHDF1* is necessary for the stable expression of *CpAphA* (Fig 7E). Consistently, the half-life time of *CpAphA* mRNA was markedly shortened in Δ*CpYTHDF1* compared with the KU80 (Fig 7F). Moreover, the mutant Δ*CpYTHDF1* exhibited a notable decrease in CpAphA protein expression, in accordance with findings on mRNA stability (Fig 7G). Together, these findings suggest that CpMTA1 regulates the *CpAphA* mRNA stability through a CpYTHDF1-dependent m^6^A modification.

### The m^6^A sites A^1306^ and A^1341^ of *CpAphA* mRNA are important for fungal phenotypic traits and virulence in *C*. *parasitica*

**Fig 8:**
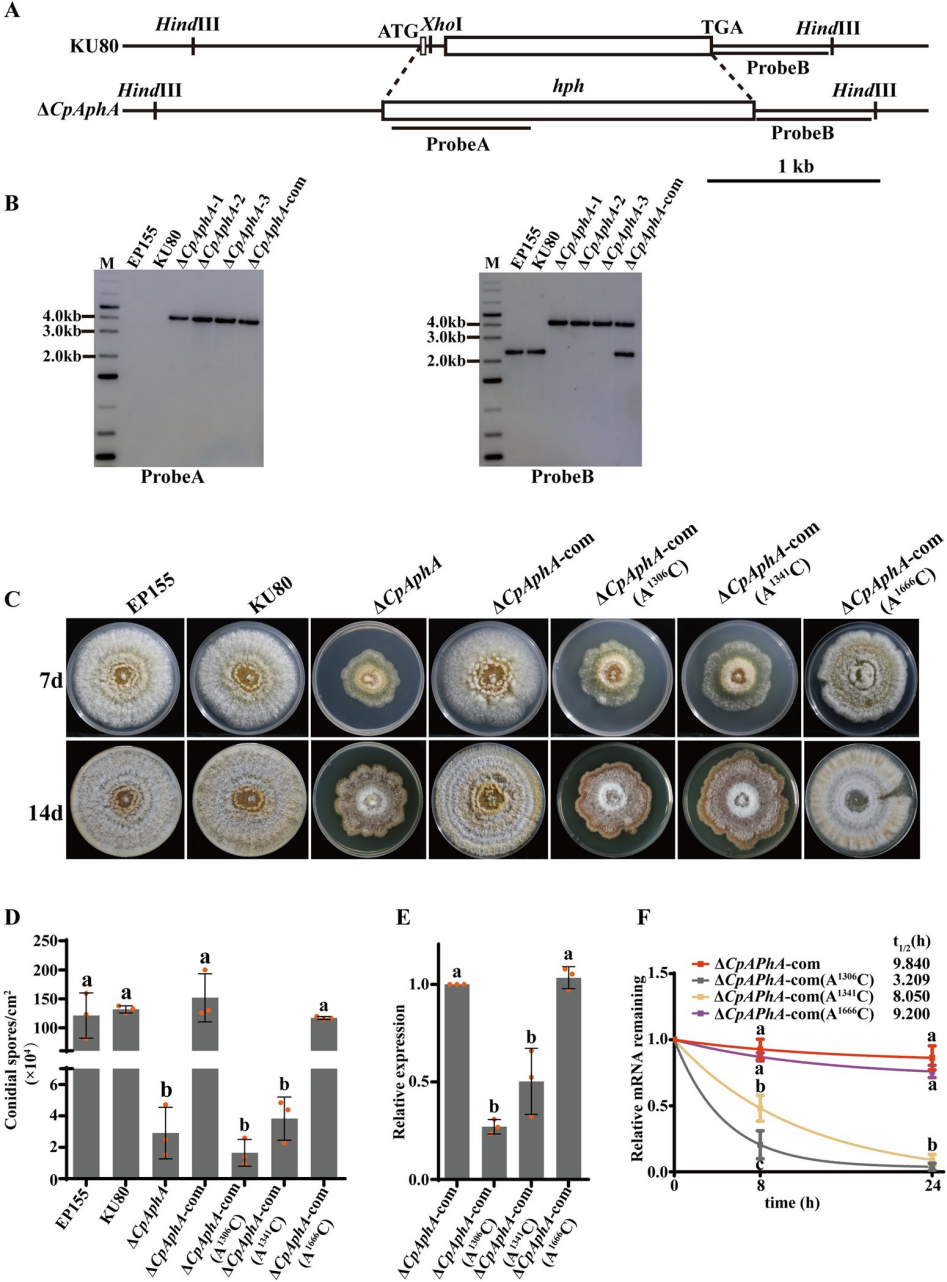
Construction and phenotype analysis of *CpAphA* mutant strains. (A): Schematic diagram of the *CpAphA* gene deletion strategy. Southern blot was performed to distinguish the wild-type strain and *CpAphA* knockout mutants using fragment on the *hph* gene (probe A) and fragment on the right arm (probe B). Scale bar, 1 kb. (B): Southern blot results of Δ*CpAphA* mutants with probe A (left) and probe B (right). Fungal total DNAs were digested with *Hind* III and *Xho* I then separated by electrophoresis, and then blotted using probe A and probe B, respectively. (C): Colony morphology of the mutants on PDA plates. Photographs were taken at 7 and 14 days post-inoculation. The wild-type EP155, parental strain KU80, *CpAphA* deletion strain Δ*CpAphA*, complementary strain Δ*CpAphA*-com, mutant complementation strains Δ*CpAphA-*com(A^1306^C), Δ*CpAphA-*com(A^1341^C) and Δ*CpAphA*-com (A^1666^C) were shown. (D): Sporulation levels were counted in the indicated strains. Spores were analyzed at 14 days post-inoculation. (E): qRT-PCR analysis of *CpAphA* mRNA expression in Δ*CpAphA*-com, Δ*CpAphA-*com(A^1306^C), Δ*CpAphA-*com(A^1341^C) and Δ*CpAphA*-com (A^1666^C). (F): The expression levels of *CpAphA* in strains were quantified by qRT-PCR at indicated time points after actinomycin D treatment and the decay rate of *CpAphA* was evaluated with a nonlinear regression model. There are significant differences between samples indicated by different letters on the bars (ANOVA followed by Tukey’s test, p < 0.05).

To further explore the effect of the m^6^A methylation site of *CpAphA* on the phenotypic traits and virulence in *C*. *parasitica*, the *CpAphA* deletion mutant was constructed firstly. The single-spored transformants were screened by PCR and confirmed by Southern blot. Further, we successfully complemented a Δ*CpAphA* mutant by re-introducing a copy of the WT *CpAphA* gene (Fig 8A and 8B). Moreover, we mutated the m^6^A methylation sites of *CpAphA* by changing A to C and transformed the mutated genes into the Δ*CpAphA* mutant, respectively. Transformants Δ*CpAphA*-com (A^1306^C), Δ*CpAphA*-com (A^1341^C) and Δ*CpAphA*-com (A^1666^C) were confirmed and subsequently used for further analysis. As shown in Fig 8C and 8D, the growth rate and sporulation of the Δ*CpAphA* mutant were decreased compared to the EP155 and KU80 strains. As expected, the abnormal phenotypes were restored in the complemented strain Δ*CpAphA*-com. The Δ*CpAphA*-com (A^1306^C) and Δ*CpAphA*-com (A^1341^C) mutants grew much more slowly on PDA plates and displayed reduced pigment and conidial spores, while the Δ*CpAphA*-com (A^1666^C) mutant showed similar phenotypes as the WT and Δ*CpAphA*-com strains. Additionally, the mutation of A^1306^C and A^1341^C resulted in a substantial decrease in mRNA expression and stability of *CpAphA*, while the mutation of A^1666^C did not (Fig 8E and 8F). Similar changes were observed in the protein level of CpAphA with western blot analysis (S12 Fig).

**Fig 9:**
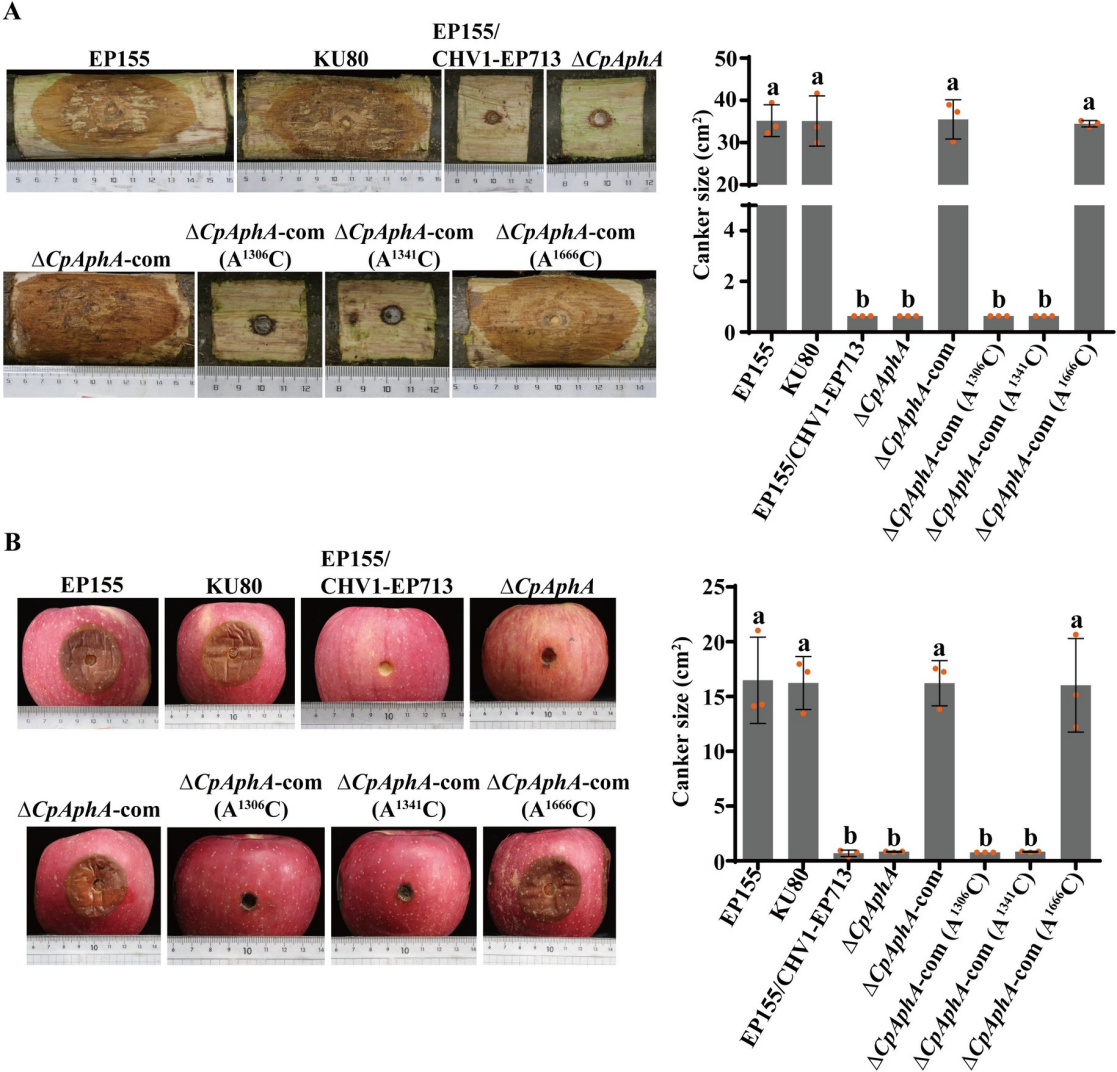
Virulence assay of *CpAphA* mutant strains. (A): Cankers were induced by the indicated strains on dormant stems of Chinese chestnut. The stems were inoculated and then maintained at 26°C, and the cankers were subsequently measured and photographed at 25 days post-inoculation. (B): Cankers were induced by the tested strains on Red Fuji apples. The inoculated apples were kept at 26°C and cankers were measured and photographed at 10 days post-inoculation. There are significant differences between samples indicated by different letters on the bars (ANOVA followed by Tukey’s test, p < 0.05).

Furthermore, the virulence assay showed the Δ*CpAphA*, Δ*CpAphA*-com (A^1306^C) and Δ*CpAphA*-com (A^1341^C) mutants incited significantly smaller cankers. The virulence of the Δ*CpAphA*-com strain showed similar virulence with the WT strain. Moreover, mutation of A^1666^C had no obvious impact on virulence (Fig 9). These results indicate that *CpAphA* is responsible for fungal phenotypic traits and virulence of *C*. *parasitica*, and the methylation sites of A^1306^ and A^1341^ play a crucial role in regulating the expression level and stability of *CpAphA in vivo*.

### Transcriptomic insight into the regulatory role of *CpAphA*

To further investigate the regulatory role of *CpAphA* in *C*. *parasitica*, RNA-seq analysis was conducted for KU80 and Δ*CpAphA* strains. Hierarchical clustering of the RNA-seq data revealed significant differences in the heat map of mRNA expression patterns between KU80 and Δ*CpAphA* (S13A Fig). A total of 965 upregulated genes and 4018 downregulated genes were identified in Δ*CpAphA* compared with KU80 (S13B Fig and S9 Table), suggesting that *CpAphA* is a global regulator gene. Additionally, KEGG enrichment analysis showed that the upregulated genes were enriched in biosynthesis of amino acids, ABC transporters, and metabolic pathways, while downregulated genes were enriched in DNA replication, cell cycle, meiosis, and mismatch repair (S13C Fig). Furthermore, GO pathway enrichment analysis indicated that upregulated genes were mostly involved in oxidation-reduction process, carboxylic acid metabolic process, integral component of plasma membrane, plasma membrane part, and oxidoreductase activity; whereas downregulated genes were significantly involved in cell cycle DNA replication, nuclear DNA replication, DNA strand elongation, nuclear replication fork, symporter activity, and sugar transmembrane transporter activity (S13D Fig).

## Discussion

**Fig 10:**
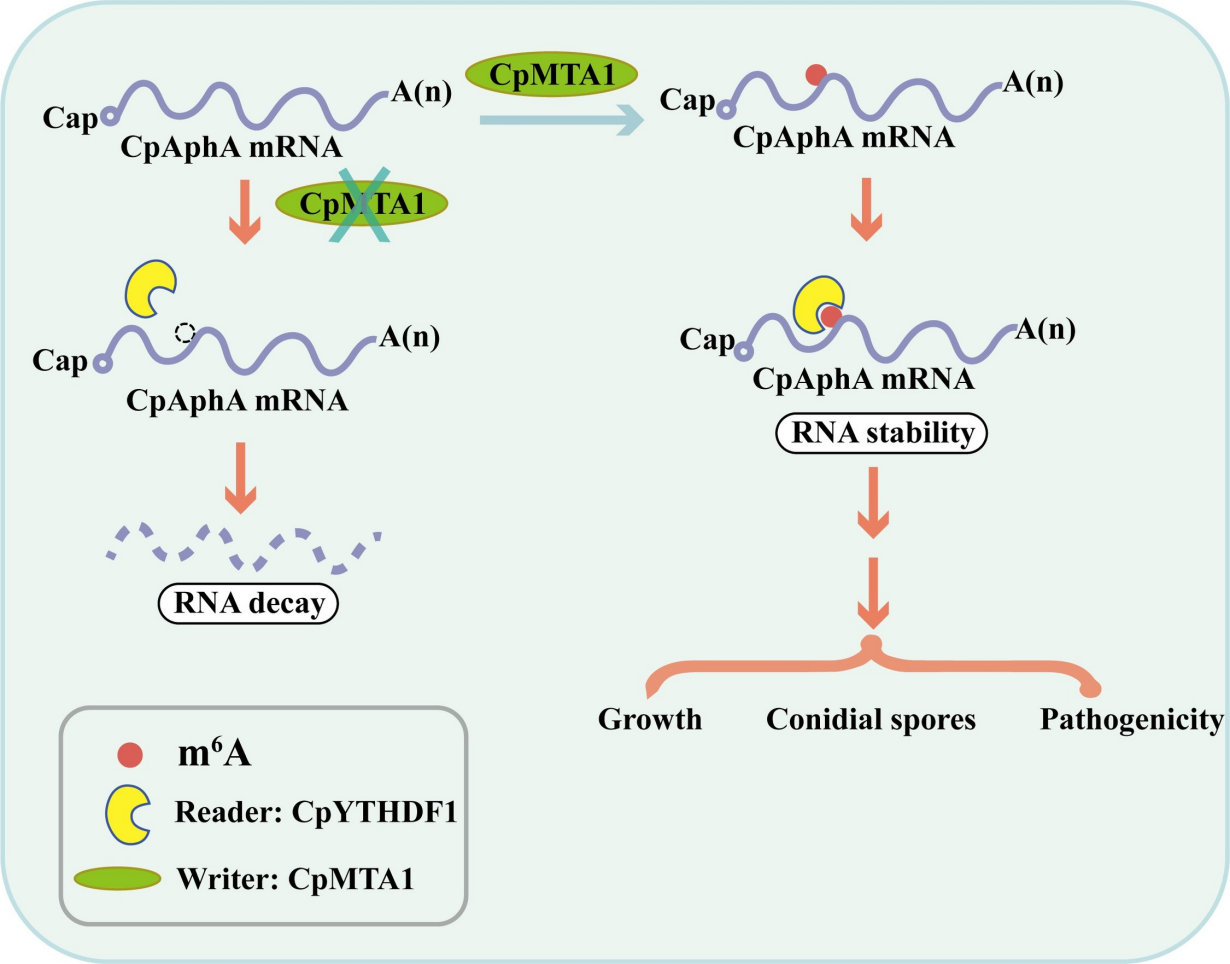
CpMTA1 mediates the *CpAphA* mRNA stability via a CpYTHDF1-mediated m^6^A modification.

Numerous modifications have been found in eukaryotic mRNA, with m^6^A appearing to be the predominant modification [41–43]. However, the biological roles and regulatory mechanisms of these m^6^A-modified enzymes are still largely unknown in filamentous fungi. In this study, we characterized an m^6^A methyltransferase, CPMTA1, in *C*. *parasitica* using a combined analysis of multiple phenotypes and multi-omics. Based on our results, CpMTA1 plays a crucial role in fungal phenotypic traits, virulence, and stress tolerance. Furthermore, integrated analysis of m^6^A-seq and RNA-seq identified *CpAphA* as a target of CpMTA1. We found that an m^6^A reader protein CpYTHDF1 could recognize *CpAphA* mRNA and increase its stability. Importantly, the m^6^A sites A^1306^ and A^1341^ of *CpAphA* mRNA are involved in the regulation of fungal phenotypic traits and virulence in *C*. *parasitica*. The schematic model shown in Fig 10 summarizes our findings.

The MT-A70 domain-containing family is considered to be the most prevalent m^6^A methyltransferases. These include Ime4 and Kar4 in yeast, as well as METTL3 and METTL14 in humans [21,44]. However, there was not found homologous proteins of human METTL3 and METTL14 in *C*. *parasitica*. Consistently, *M*. *oryzae* MTA1, exhibits orthology with human METTL4, not METTL3 [5]. Recently, *Fusarium graminearum* was discovered to contain MTA1, a homolog of METTL4, as a sole MT-A70-containing protein. Furthermore, species within *Pezizomycotina*, which is comprised entirely of filamentous fungi, may have experienced the loss of the m^6^A writers Ime4 and Kar4. In contrast, budding yeasts (*Saccharomycotina*) may have undergone the loss of the m^6^A writer MTA1 [45]. Here, we showed that the CpMTA1 protein has the conserved MT-A70 domain. Phylogenetic analysis also found that CpMTA1 is much more closely related to *M*. *oryzae* MoMTA1 and human METTL4 than to METTL3 (S1 Fig). As a subclade of the MT-A70 family, METTL4 is separated from the METTL3 and METTL14 subclades. Despite METTL4 being reported to be a U2 snRNA N^6^-adenosine methyltransferase in *Drosophila* and human [12], it is not clear whether METTL4 homologs can regulate m^6^A methylation of mRNA. Here, we found that the deletion of the *CpMTA1* led to a notable reduction in overall m^6^A levels (Fig 1), and the m^6^A-seq analysis revealed that 3285 KU80-unique m^6^A peaks were lost in Δ*CpMTA1* mutant (Fig 4), suggesting an important role for CpMTA1 in the m^6^A modification of mRNA in *C*. *parasitica*.

Our results found that the knockout strain Δ*CpMTA1* still retains some level of m^6^A methylation (Fig 1), consistent with the result reported in *M*. *oryzae* [5]. Similarly, depletion of METTL3 and METTL14 is known to cause a ~60% reduction in m^6^A modification in some cell types [46]. These observations suggest the presence of additional m^6^A methyltransferases. More recently, METTL16 has also been identified as an m^6^A methyltransferase. Originally thought to target only a few transcripts, including 3 noncoding RNAs (U6 snRNA, *MALAT1* and *XIST*) and one mRNA (*MAT2A*), METTL16 has now been shown to function as an m^6^A writer to deposit m^6^A into hundreds of specific mRNA targets [47]. It is a conserved protein with homologs found from vertebrates to yeast and bacteria [48]. Using human METTL16 as a query, we also identified a homologous protein XP_040778198 in *C*. *parasitica*, named CpMETTL16. Our preliminary results showed that knockout of *CpMETTL16* gene resulted in a significant decrease in m^6^A abundance. It would also be interesting to investigate if CpMETTL16 could compensate for activity in Δ*CpMTA1* in further studies.

Previous studies have reported that loss of the m^6^A methyltransferase can affect stem cell differentiation, animal and plant development, viral infection, spermatogenesis, sex determination, stress response, and cancer development [49]. The disruption of the *MTA1* gene has a significant impact on vegetative growth, conidial formation, appressorium and pathogenicity in *M*. *oryzae* [5]. However, extensive efforts to generate homozygous *MTA1* knockout were unsuccessful, indicating its essential function in *F*. *graminearum* [45]. In this study, the homozygous Δ*CpMTA1* mutant was viable and showed a significant decrease in growth rate, sporulation, virulence, and stress tolerance (Figs 2 and 3). Because severe growth retardation of the *CpMTA1* knockout strains was found, decreases in the virulence of Δ*CpMTA1* may simply result from the growth defect. Therefore, if virulence determinants are defined as factors that exclusively impact virulence, *CpMTA1* may not be considered a specific virulence factor but rather a vital gene for normal fungal growth and development. Consistently, the gene expression profile showed significant changes in response to the deletion of *CpMTA1*. Specifically, the loss of CpMTA1 resulted in a decrease in 854 gene expressions and an increase in 727 gene expressions (Fig 5). Among these genes, CpAphA was identified as a crucial target of CpMTA1. While our focus was on the regulatory role of *CpAphA*, it is possible that other target genes also contribute to fungal development and virulence of Δ*CpMTA1*. One of the downregulated genes was Glycoside hydrolase family 12 (xgeA), which has been linked to reduced virulence in *Fusarium oxysporum* when deleted or its enzyme activity lost [50]. Two other noteworthy differentially expressed genes are subtilisin-like protein (aorO) and citrate synthase (gltA). Previous reports have demonstrated that Prb1, a subtilisin-like protease, is essential for virulence and phenotypical traits in *C*. *parasitica* [51]. Additionally, deletion of citrate synthase led to a significant reduction in growth rate, sporulation, and virulence of *C*. *parasitica* [32], resembling the phenotype observed with Δ*CpMTA1*.

By m^6^A-seq analysis, we revealed that m^6^A peaks of *C*. *parasitica* were significantly enriched in RRACH and DRACH, which are the conserved m^6^A motif among mammals [52,53]. In comparison, m^6^A modifications in plants involve complicated sequence preferences, such as RRACH, URUAH, AAACCV (V means U, A, or G), and WKUAH (W means U or A; K means G or U) motifs [54]. The transcript-specific m^6^A localization has also been investigated at the transcriptome-wide level in *C*. *parasitica*. Our data showed that m^6^A peaks in *C*. *parasitica* transcriptome were abundant in the CDS region and near stop codons (Fig 4). Though m^6^A distribution around the stop codon or in the 3′ UTR are conserved among various plants and mammals, m^6^A deposition in the CDS region can also be found in some plants [54]. The m^6^A modification in strawberry are highly enriched in the CDS region adjacent to the start codon, besides the occurrence in the stop codon and 3′ UTR region [55]. Moreover, m^6^A modifications in pak-choi leaves and apple are most abundant in the CDS region, followed by the 3′ UTR region [56,57]. Therefore, the m^6^A distribution appears to differ among different species, suggesting that m^6^A modifications can mediate all stages of the mRNA life-cycle.

Correlation analysis of m^6^A-seq and RNA-seq data suggested that the m^6^A methylation level was significantly positively correlated with the gene expression level in *C*. *parasitica* (Fig 5). This observation was consistent with some previous studies, while it was different from others [58]. The difference may be attributed to the species types and collection of samples. For instance, m^6^A within the 3’ UTR or near the stop codon exhibits the capacity to decrease mRNA stability in normally growing maize seedling, tomato fruit, and strawberry fruit, while m^6^A enriching in the CDS region tends to increase mRNA stability in ripe strawberry fruit [59]. Coincidentally, the mRNA expression and stability of *CpAphA* were decreased in the Δ*CpMTA1* mutant, proving that the m^6^A methylation positively mediates mRNA abundance.

Importantly, m^6^A modification can also influence RNA fate by m^6^A readers, which preferentially recognize and bind modified nucleotides. Currently, various m^6^A reader proteins have been reported in mammals, including the YTHDF family, YTH domain containing proteins (YTHDC1/2), IGF2 mRNA binding proteins (IGF2BP1/2/3), eukaryotic initiation factor 3 (eIF3), and heterogeneous nuclear ribonucleoproteins (HNRNP) family [60]. Among them, the YTHDF family proteins are the most studied m^6^A readers, located in the cytoplasm including YTHDF1, YTHDF2, and YTHDF3. By influencing translation and stability of target mRNAs, they affect the expression of downstream molecules, as well as various biological processes. However, the role of each protein is different, YTHDF1 improve RNA translation, and further promote its RNA stability, YTHDF2 facilitates RNA decay and YTHDF3 has a bidirectional regulation effect [61]. To identify the YTHDF homologous proteins in *C*. *parasitica*, the amino acid sequence of human YTHDF1/2/3 were used as a query for the BLASTp similarity search against the *C*. *parasitica* genome, respectively. Only one m^6^A reading protein was identified, which is homologous to human YTHDF1. Our data showed that the m^6^A reader protein CpYTHDF1 could recognize *CpAphA* mRNA, and further promote its RNA stability, which is consistent with the function of YTHDF1 in mammals. In contrast, YTH1 and YTH2 proteins that contain a YTH domain have been characterized in *M*. *oryzae* [23]. Protein sequence alignment confirmed that *C*. *parasitica* CpYTHDF1 is indeed closer to *M*. *oryzae* YTH1, but far from YTH2. Because m^6^A reading proteins in fungi are rarely reported, further studies are needed to demonstrate the function and regulatory mechanism of these proteins.

It was observed that the deletion of *CpAphA* gene resulted in a decrease in growth rate, sporulation, and pathogenicity, which was consistent with the *ΔCpMTA1* strain (Fig 8). Therefore, CpMTA1 may regulate *C*. *parasitica* growth and virulence by downregulating *CpAphA* expression. *CpAphA* was annotated to a metallo-dependent phosphatase (XP_040780407.1) by NCBI. RNA-seq analysis of *ΔCpAphA* suggested that *CpAphA* is a global regulator through influencing multiple genes (S13 Fig). Previous evidence also indicates important functions of individual metallo-dependent protein phosphatases (PPM) isoform in signalling and cellular processes, including senescence, proliferation, apoptosis, and metabolism in animals [62] and plants [63]. Nevertheless, there is a lack of report regarding the regulatory effects of m^6^A modification on phosphatase protein. Further analysis of the detailed regulatory mechanisms of *CpAphA* will facilitate our understanding of the role of protein phosphatases in fungi.

Moreover, the methylation sites of A^1306^ and A^1341^ of *CpAphA*, which are located in CDS region, were found to be essential for regulating the expression level and stability of *CpAphA in vivo*, but not in the 3’ UTR region (A^1666^) (Fig 8). After analyzing the amino acid sequence, we found that the mutation at position A^1306^C did not change its corresponding amino acid, while the mutation at position A^1341^C resulted in the substitution of Asn^415^ with Thr. Through the functional domain and active site analysis of CpAphA protein (S11 Fig), we found that Asn^415^ was located in the MPP-PAP domain, but not in the active site. Together, these data suggest that the mutation of A^1306^ affects mRNA deposition rather than protein function, while the mutation of A^1341^ may affect mRNA deposition and protein function. Meanwhile, previous studies have reported that m^6^A “readers” are required in m^6^A-regulated diverse downstream signaling pathways [61]. Although our data showed that the m^6^A reader protein CpYTHDF1 could recognize *CpAphA* mRNA, the exact binding site has not been determined currently. We hypothesized that CpYTHDF1 does not recognize the m^6^A-modified A^1666^ site, so mutating this site does not change the fate of *CpAphA* mRNA. It remains to be determined which specific regions of CpAphA mRNA are recognized by m^6^A reader in *C*. *parasitica*.

To our knowledge, this is the first study to demonstrate that *CpAphA* is a direct downstream target of CpMTA1-mediated m^6^A modification, revealing the mechanisms by which CpMTA1 can manipulate *CpAphA* and regulate fungal phenotypic traits and virulence. Together, the present findings provide a comprehensive insight into the function and mechanism of CpMTA1-mediated m^6^A modification in filamentous fungi, and expand the understanding of related studies.

## Materials and methods

### Fungal strains and culture conditions

Wild-type (WT) *C*. *parasitica* strain EP155 (ATCC 38755), its isogenic hypovirulent strain EP155/CHV1-EP713 (hypovirus CHV1-EP713 infected EP155, ATCC 52571)

[64], the highly efficient gene deletion strain KU80 (Δ*cpku80* of EP155) [65], and mutant strains were cultured on potato glucose agar (PDA, Difco) medium at 26°C with a 12 h light/dark cycle [66] for phenotypic analyses, DNA and RNA extraction. For protein extraction, liquid EP complete medium was used as described previously [67].

### Construction of fungal mutants

The knockout of *CpMTA1* gene was performed through homologous recombination method as described previously [65]. The upstream (983 bp) and downstream (927 bp) sequences of the *CpMTA1* gene and the hygromycin B resistance marker (2145 bp) were amplified from *C*. *parasitica* genome DNA and plasmid pCPXHY2, respectively. These three fragments were joined by overlap extension PCR and the resulting fused cassette was transformed into KU80 strain using PEG-mediated protoplast transformation. The positive transformants were identified by PCR, RT-PCR and Southern blot analysis, followed by single-spore isolation to achieve nuclear homogeneity [68]. To complement the *CpMTA1* knockout mutant strain, the entire open reading frame (ORF) of *CpMTA1* gene, along with its promoter, was amplified using PCR and subsequently inserted into the pCPXG418 vector, which harboured the geneticin resistance gene (G418) [69]. The resulting vector pCPXG418-*CpMTA1* was transformed into the knockout strain Δ*CpMTA1*. Complemented strains were then confirmed by PCR, qRT-PCR and Southern blot analysis. The Mut Express II fast mutagenesis kit V2 (Vazyme Biotech) was used to construct point mutation of CpMTA1 with pCPXG418-*CpMTA1* as the template. Each mutant vector was verified by DNA sequencing and transformed into Δ*CpMTA1*.

To study the subcellular localization of CpMTA1, the CpMTA1 ORF was inserted into pCPXG418-GFP vector, and the recombinant plasmid pCPXG418-CpMTA1-GFP was transformed into Δ*CpMTA1*. A positive GFP-tagged strain was chosen for subcellular localization analysis on an Olympus BX51 fluorescence microscope. The construction of gene deletion, complementation, and point mutation of the *CpAphA* gene were performed using similar methods described above.

To construct strains expressing N-terminally 3×flag-tagged CpMTA1 and CpYTHDF1 fusion protein, CpMTA1 ORF and CpYTHDF1 ORF were cloned into a modified pCPXG418-3×flag vector, respectively. The correct pCPXG418-3×flag-CpMTA1 and pCPXG418-3×flag-CpYTHDF1 plasmid were transformed into the WT EP155 strain separately, and the expression of CpMTA1 and CpYTHDF1 was validated by PCR and western blotting. The construction of strains expressing C-terminally 3×flag-tagged CpAphA fusion protein were performed with a similar method. S10 Table lists the primer sequences used in this study.

### Detection of methylation level of m^6^A RNA

Total RNA was extracted from *C*. *parasitica* by the MiniBEST Plant RNA Extraction Kit (Takara). The methylation level of m^6^A RNA was detected with the colorimetric EpiQuik m^6^A RNA methylation quantification kit (Epigentek, USA) according to the manufacturer’s protocol. After coating 200 ng RNA and the m^6^A standard onto the assay wells, capture and detection antibody solutions were sequentially added. Using a wavelength of 450 nm (OD_450_), the absorbance of each well was measured to estimate m^6^A levels. The relative m^6^A RNA methylation levels were then calculated.

### m^6^A dot blot assay

Total RNA was extracted from the KU80, Δ*CpMTA1*, Δ*CpMTA1*-com and Δ*CpMTA1* (D158A/W161A), and spotted onto a Hybond-N+ membrane. The sample volume used was 1.5 μL, followed by UV crosslinking for 3 minutes at a strength of 1500 mJ/cm^2^ after drying the membrane. Subsequently, the membrane was blocked in 5% milk phosphate buffered saline Tween (PBST) for 1 h before being incubated with an m^6^A antibody (EpiGentek) overnight at 4°C. After washing with PBST, the membrane was incubated with the secondary antibody for 1 h at room temperature. Finally, visualization was carried out using the ECL detection system (AI600 images).

### Fungal phenotype and virulence analysis

The WT strain EP155, KU80, and EP155/CHV1-EP713 were compared to the constructed mutant strains in terms of phenotypic and virulent properties. Phenotypic changes in growth rate, conidiation and pigmentation were analyzed as previously described [31]. After the fungi were cultured on PDA for 14 days, the conidia from each sample were eluted and counted with a hemacytometer. To test the stress tolerance, PDA was used as the base medium supplemented with different chemical reagents (H_2_O_2_, SDS, NaCl and Cogo Red). To detect fungal virulence, the dormant Chinese chestnut (*Castanea mollissima*) stems or Red Fuji apple were inoculated with strain samples, respectively. Stems or apples were incubated at 26°C for 25 days or 10 days to allow lesion development. Finally, canker size was observed and analyzed [68].

### m^6^A-seq analysis

The *C*. *parasitica* strain KU80 and Δ*CpMTA1* were used for m^6^A-seq analysis, and three biological replicates were used for each strain. RNA was prepared from mycelial mats grown on PDA at 7 d post-inoculation. A total of 10 μg RNA was extracted for each sample and fragmented into short fragments (about 100 nt) using fragmentation buffer. A portion of untreated fragmented RNA was used for RNA-seq as input control. Another portion of fragmented RNA and anti-m^6^A antibody were incubated at 4°C for 2 h in IP buffer. Next, cDNA libraries were constructed from m^6^A-containing fragments (IP) and untreated input control fragments. The cDNA fragments were ligated to Illumina sequencing adapters by paired-end strategy. The m^6^A-seq and input RNA-seq were performed using Illumina HiSeq 4000 of Gene Denovo Biotechnology Co (Guangzhou, China).

The IP RNA-seq and input RNA-seq raw data were filtered by Fastp software (version 0.18.0), removing reads containing adapters and low-quality bases (Qvalue≤20) [56]. With HISAT2. 2.4 software [70], clean data from the IP and input samples were mapped to the *C*. *parasitica* reference genome (https://genome.jgi.doe.gov/portal/Crypa2/Crypa2.download.ftp.html). According to the background information (input RNA-seq data), exomePeak2 software [71] was used to identify m^6^A peaks with default settings. PeakAnnotator (version 2.0) was performed to annotate m^6^A peaks to the *C*. *parasitica* annotation file [72]. The exomePeak2 software was used to identify the differentially methylated peaks between the KU80 and Δ*CpMTA1* with a criterion of log_2_|FC|≥1 and FDR≤0.05.

### RNA-seq analysis

The input sequencing reads from m^6^A-seq were used for RNA-seq analysis as described previously [73]. Using StringTie v1.3.1, mapped reads were assembled in a reference-based approach for each sample [74]. The FPKM (fragment per kilobase of transcript per million mapped reads) value was calculated to quantify gene expression level and variations by RSEM software [75]. Significant DEGs analyse were determined using DESeq2 [76], and defined by FDR≤0.05 and log_2_|FC|≥1 in the two groups.

### Quantitative real-time PCR

Total RNA was extracted from the strain samples using MiniBEST Plant RNA Extraction Kit (Takara), and then cDNA was produced using HiScript III RT SuperMix (Vazyme Biotech, R323). With the SYBR Green PCR Master Mix (Takara), quantitative real-time PCR (qRT-PCR) was performed on the LightCycler480II real-time PCR system (Roche).

### Methylated RNA immunoprecipitation coupled with qRT-PCR

For methylated RNA Immunoprecipitation (MeRIP)-qPCR, the m^6^A RNA Enrichment Kit (Epigentek, USA) was used according to the manufacturer’s instructions. A total of 10 μg RNA was extracted for each sample, and reduced into fragments of 300 or fewer nucleotides. Magnetic beads containing 10 μg anti-m^6^A antibody or IgG were used to immunoprecipitate RNA samples. The IP or input assay products were then analyzed with qRT-PCR using primers (S10 Table).

### RNA restriction enzyme MazF assay

RNase MazF (TaKaRa 2415A) was used to confirm the methylation site on *CpAphA* mRNA as described previously [77]. Here, 12.5 μg of total RNA was subjected to isolate poly(A) mRNA using VAHTS mRNA Capture Beads (Vazyme) and mixed with 4 μL MazF buffer, 0.5 μL RNase inhibitor and 1 μL MazF (TaKaRa). Then the 20 μL mixture was incubated at 37°C for 2 h. Next, RNA was resuspended in 12 μL RNase-free water, and cDNA was synthesized by HiScript III RT SuperMix (Vazyme Biotech, R323) kit. The resulting cDNA was employed in subsequent PCR reactions with special primers (S10 Table), and the products were analyzed by a TBE gel.

### Western blot

Total fungal proteins were extracted with NP40 lysis buffer (Solarbio) as described previously [78]. After boiled with SDS loading buffer, the protein was separated using 12% SDS-PAGE and transferred to a PVDF membrane (Millipore, USA). Next, the membrane was blocked and incubated with anti-flag antibody (Abmart) or anti-β-actin antibody (ABclonal) at 4°C overnight. Finally, the membrane was detected using an ECL detection reagent (Coolaber, China).

### RNA immunoprecipitation

The RIP assay was performed using the RNA Immunoprecipitation (RIP) Kit (BerSinBio, Bes5101) based on the provided directions. Briefly, the collected fungal cells were lysed with RIP lysis buffer on ice, and DNase was used to remove DNA. Then the cell lysate was immunoprecipitated with anti-flag antibody (1:100) at 4°C for 16 h, and incubated with protein A/G beads for 1 h. Finally, co-precipitated RNAs were detected using qRT-PCR. The primers used in this assay are shown in S10 Table.

### mRNA stability detection

To determine the *CpAphA* mRNA stability in KU80 and Δ*CpMTA1* strains, actinomycin D (GlpBio Technology, CA, USA) was added to the EP liquid medium to the final concentration of 20 μM. The mock control used was DMSO. At the indicated time point of 0 h, 8 h, and 24 h, fungal samples were collected for RNA extraction. *CpAphA* mRNA expression was detected by qRT-PCR [79].

### Statistical analysis and reproducibility

All statistical differences among the samples were carried out by Tukey’s test and analysis of variance using IBM SPSS Statistics 22 software. All data are shown as mean ± SD with error bars. A p-value < 0.05 was considered statistically significant. Unless otherwise noted, all experiments were repeated three times.

## Supporting information

S1 Data Excel spreadsheet containing, in separate sheets, the underlying numerical data and statistical analysis for Figs 1D, 2B, 2C, 3A, 3B, 5B, 6D, 6E, 6H, 7A, 7D, 7E, 7F, 8D, 8E, 8F, 9A, 9B, S6 and S10. (XLSX)

S1 Fig Phylogenetic analysis, conserved domain, and sequence similarity comparison of CpMTA1 homologues from different organisms. (A): Phylogenetic tree of MT-A70 domains orthologs from diverse species using MEGAX software analysis. (B): Conserved domain of CpMTA1 homologous proteins. The structural domains of these sequences were analyzed using the NCBI website (https://www.ncbi.nlm.nih.gov/Structure/bwrpsb/bwrpsb.cgi) and TBtools software. (C): Comparison of sequence similarity between CpMTA1 and other homologous proteins. The sequence similarity between CpMTA1 and other homologous proteins was determined using DNAMAN software.
(TIF)

S2 Fig Construction and verification of Δ*CpMTA1-*com. (A): Schematic diagram of the construction of *CpMTA1* gene complement plasmid pCPXG418-com-*CpMTA1*. (B): Verification of the plasmid pCPXG418-com-*CpMTA1* using *EcoR* I/*Not* I digestion. 1: The *CpMTA1* gene. 2: The pCPXG418 plasmid after *EcoR* I/*Not* I digestion. 3: The pCPXG418*-*com-*CpMTA*1 plasmid. 4: The pCPXG418-com-CpMTA1 plasmid after *EcoR* V/ *Not* I digestion.
(TIF)

S3 Fig Analysis of conserved sites of CpMTA1. (A): Prediction of conserved active sites by InterPro. (B): The sequence alignment of CpMTA1 and its orthologs was performed using CLC Genomics Workbench. The red boxes represent the conserved sites of CpMTA1.
(TIF)

S4 Fig The structure of CpMTA1 and its mutant protein was predicted using AlphaFold2. Asp-158, Pro-159, Pro-160, and Trp-161 are highlighted in red (left). To analyze the amino acid residues at positions 158 and 161 before and after mutation, PyMOL v2.5.4 was used to create a hydrogen bond interaction map. Amino acids 158 and 161 are shown in blue, and hydrogen bonds are represented by yellow dashed lines (right).
(TIF)

S5 Fig The quality of predicted structure of CpMTA1 protein. (A): Ramachandran plot of the predicted structure of wild-type CpMTA1 protein. (B): Ramachandran plot of the predicted structure of mutated CpMTA1 protein (APPA). (C): The predicted CpMTA1 structure by AlphaFold2 was compared with Arabidopsis METTL4 structure (PDB DOI: https://doi.org/10.2210/pdb7CVA/pdb) using PyMOL software. The root mean square deviation (RMSD) is 1.255, indicating that the similarity between the two proteins (Arabidopsis METTL4 is shown in red, CpMTA1 is shown in green).
(TIF)

S6 Fig Fungal strains were grown on PDA medium containing H_2_O_2_, SDS, NaCl, or Congo red. (A): Colonies of the wild type, deletion mutant, complementation mutant and overexpression mutant were shown after 7 days of cultivation at 26°C. (B): Growth inhibition rate of strains by stressors, and colony diameter on PDA was set to 100%. All measurements were performed after 7 days of growth at 26°C and were performed in triplicate. Error bars represent the standard deviation. Different letters on the bars indicate significant differences (p < 0.05).
(TIF)

S7 Fig Colony morphology and viral RNA accumulation were examined in hypovirus-containing Δ*CpMTA1* mutants. (A): The colony morphology of hypovirus-free and hypovirus containing Δ*CpMTA1* mutants was observed on PDA at day 7 post-inoculation. (B): Viral dsRNA accumulation was analyzed using agarose gel electrophoresis in hypovirus-containing Δ*CpMTA1* mutants.
(TIF)

S8 Fig Gene set enrichment analysis (GSEA) of the potential downstream target genes of *CpMTA1*. The top of the graph represents the enrichment score (ES): The ES value reflects the degree to which the members of the gene set are enriched at both ends of a sorted list. A positive ES indicates that the gene set is enriched at the top of the list, i.e., the pathway is activated or upregulated; while a negative ES indicates that the gene set is enriched at the bottom, i.e., the pathway is repressed or downregulated. The color of the curve is indicative of the color associated with the KEGG pathway listed on the right. The bottom of the graph shows the distribution of the rank values of all genes after sorting.
(TIF)

S9 Fig Analysis of m^6^A modification in the KU80 and Δ*CpMTA1* mutant strains. (A): Distribution of m^6^A peaks along the whole mRNA transcripts of *C*. *parasitica* detected in the KU80 strain and the Δ*CpMTA*1 mutant. (B): Number of the m^6^A-modified transcripts containing different m^6^A peak numbers in the KU80 strain and the Δ*CpMTA1* mutant.
(TIF)

S10 Fig RIP assay detected the association between CpMTA1 and potential target genes. RIP assay was performed using an anti-flag antibody in EP155/3×flag-CpMTA1 strain. β-actin was used as a negative control. The fold enrichment values were normalized to that of Input. The same letters on the bars mean no significant difference between samples (ANOVA followed by Tukey’s test).
(TIF)

S11 Fig Analysis of conserved domains of CpAphA protein in NCBI. (TIF)

S12 Fig Expression of CpAphA protein was verified by western blotting using an anti-flag antibody. Anti-β-actin antibody was used for the control. (TIF)

S13 Fig RNA-seq analysis identified transcripts affected by *CpAphA* deletion. (A): Heatmap showing relative expression levels of genes in Δ*CpAphA* relative to KU80 strain based on the RNA-seq data. Cutoff criteria for differentially expressed genes included log_2_|FC|≥1 and FDR≤0.05 (red color indicates an increase and blue color represents a decrease in mRNA expression.) (B): Volcano plot showing the DEGs between KU80 and Δ*CpAphA*. (C): KEGG pathway enrichment analysis of DEGs. (D): GO-based enrichment analysis of DEGs in terms of biological process (BP), cell component (CC) and molecular function (MF).
(TIF)

S1 Table All m^6^A peaks of transcripts identified by m^6^A-seq. (XLSX)

S2 Table GSEA analysis of genes with unique m^6^A peak in KU80. (XLSX)

S3 Table Distribution of m^6^A peaks along the whole mRNA transcripts. (XLSX)

S4 Table Differentially expressed genes in KU80 and Δ*CpMTA1*. (XLSX)

S5 Table Genes with a significant change in both m^6^A level and mRNA expression in *CpMTA1* deletion strain compared to the control KU80. (XLSX)

S6 Table Analysis of the genes with unique m^6^A peak in KU80 and down-regulated expression in Δ*CpMTA1*, or up-regulated expression in Δ*CpMTA1*. (XLSX)

S7 Table KEGG pathway enrichment analysis of the genes with unique m^6^A peak in KU80 and down-regulated expression in Δ*CpMTA1*. (XLSX)

S8 Table The number of m^6^A peaks on genes with unique m^6^A peak in KU80 and down-regulated expression in Δ*CpMTA1*. (XLSX)

S9 Table Differentially expressed genes in KU80 and Δ*CpAphA*. (XLSX)

S10 Table Primers used in this study. (XLSX)
