# Supplementary figures and images for: N6-methyladenosine RNA methyltransferase CpMTA1 mediates CpAphA mRNA stability through a YTHDF1-dependent m6A modification in the chestnut blight fungus
Source: PLoS Pathog. 2024 Aug 19;20(8):e1012476. doi: 10.1371/journal.ppat.1012476 (PMC11361730; doi:10.1371/journal.ppat.1012476)

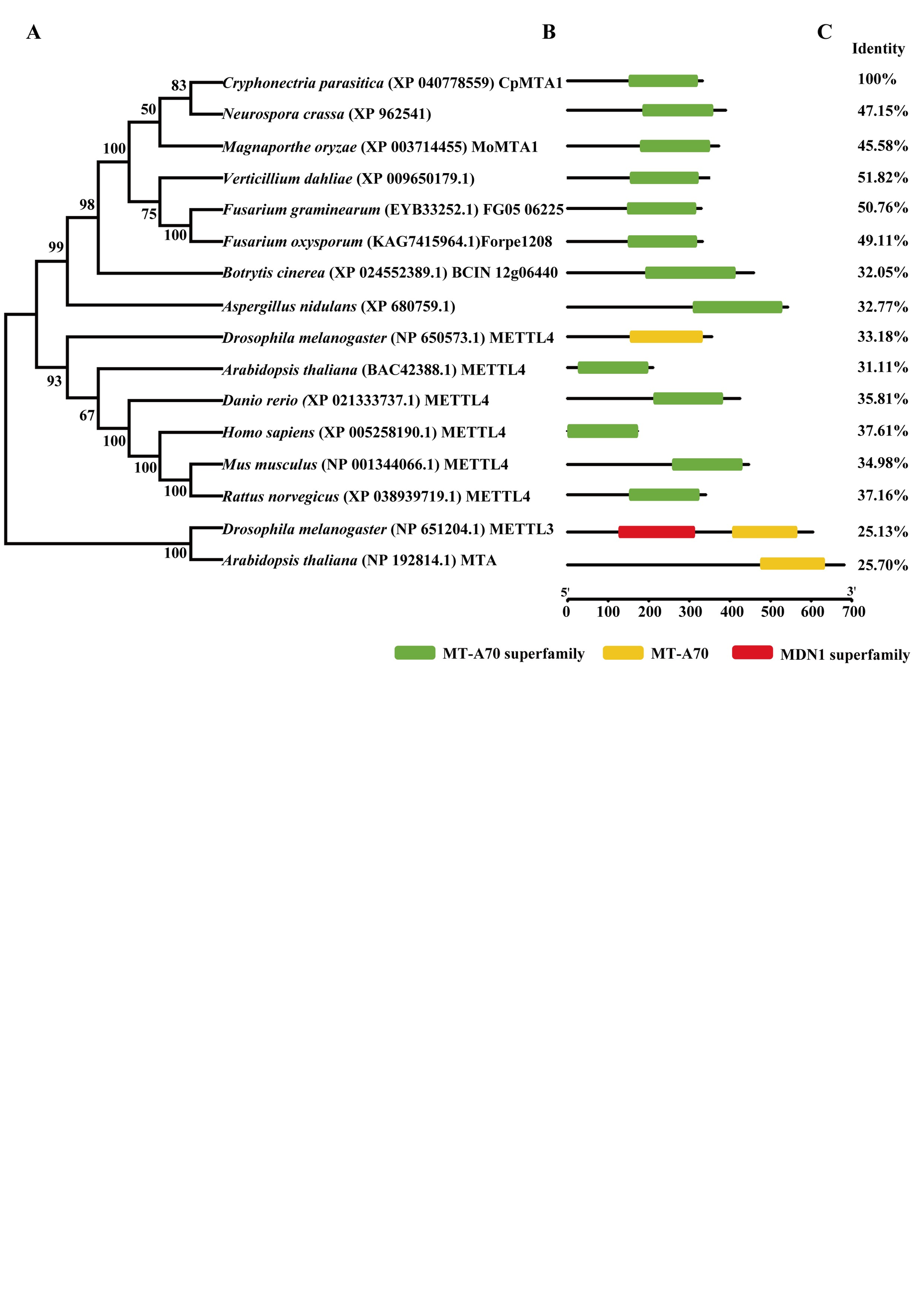

Supplement: S1 Fig — (A): Phylogenetic tree of MT-A70 domains orthologs from diverse species using MEGAX software analysis. (B): Conserved domain of CpMTA1 homologous proteins. The structural domains of these sequences were analyzed using the NCBI website (https://www.ncbi.nlm.nih.gov/Structure/bwrpsb/bwrpsb.cgi) and TBtools software. (C): Comparison of sequence similarity between CpMTA1 and other homologous proteins. The sequence similarity between CpMTA1 and other homologous proteins was determined using DNAMAN software. (TIF) [file ppat.1012476.s002.tif]

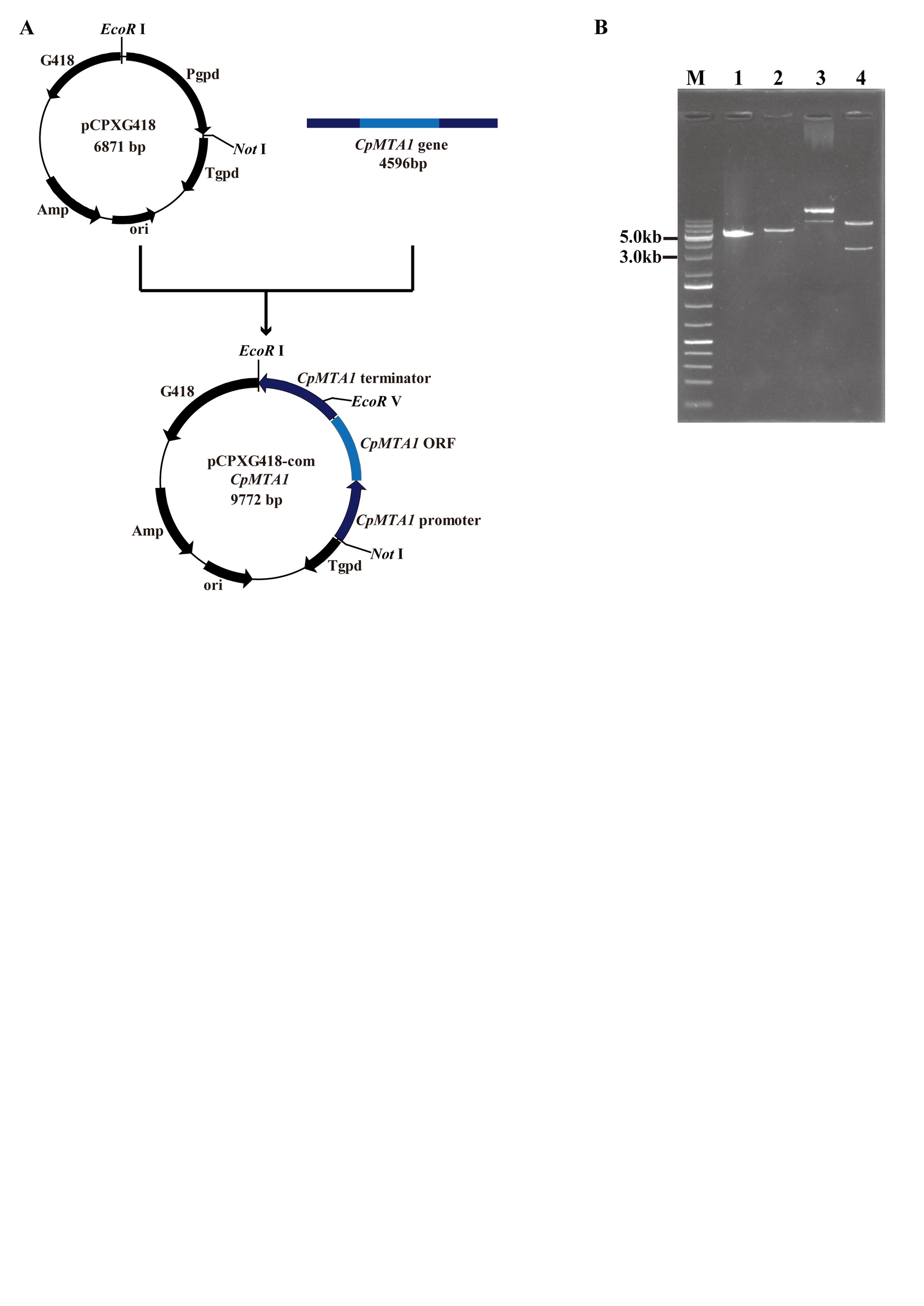

Supplement: S2 Fig — (A): Schematic diagram of the construction of CpMTA1 gene complement plasmid pCPXG418-com-CpMTA1. (B): Verification of the plasmid pCPXG418-com-CpMTA1 using EcoR I/Not I digestion. 1: The CpMTA1 gene. 2: The pCPXG418 plasmid after EcoR I/Not I digestion. 3: The pCPXG418-com-CpMTA1 plasmid. 4: The pCPXG418-com-CpMTA1 plasmid after EcoR V/ Not I digestion. (TIF) [file ppat.1012476.s003.tif]

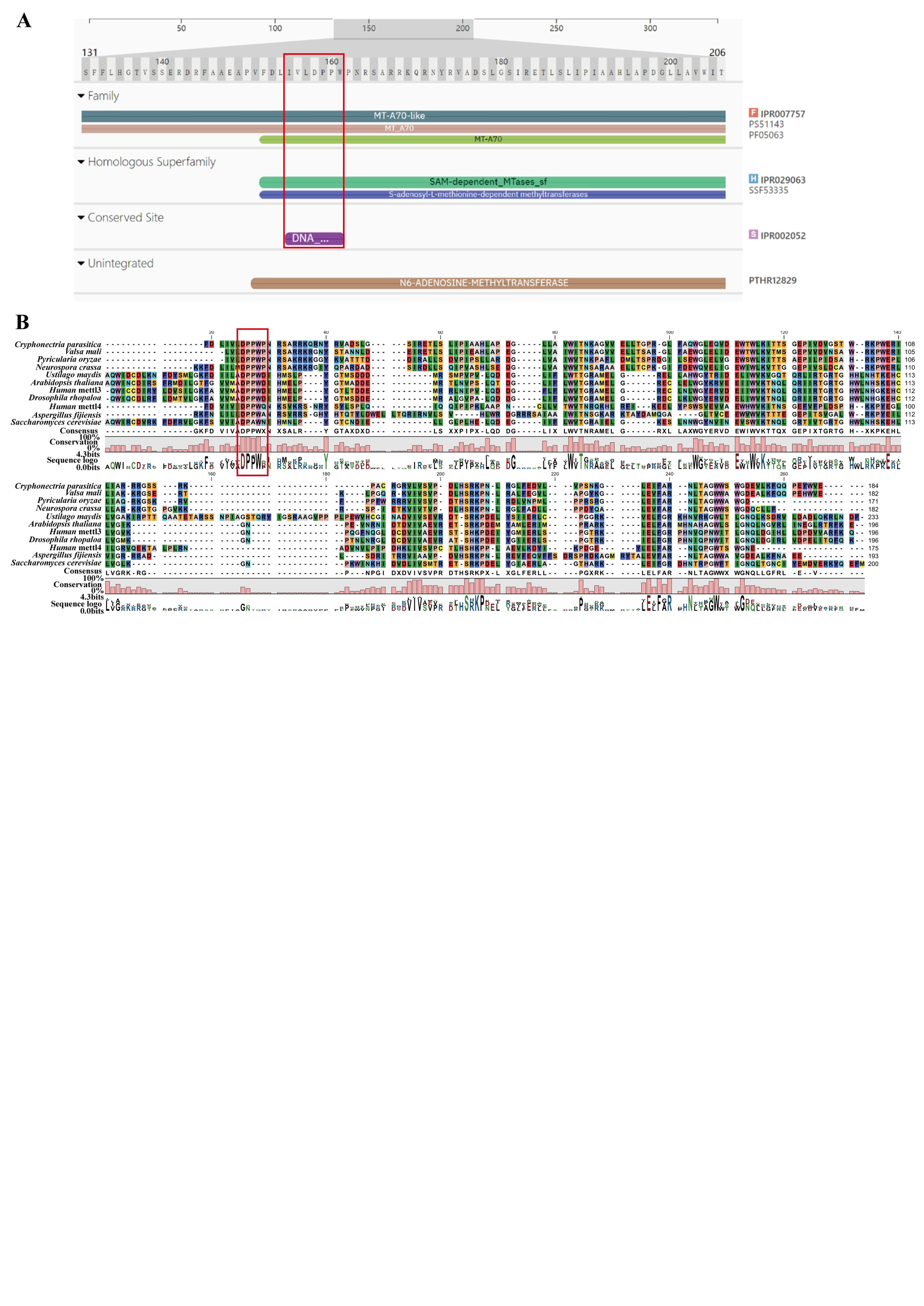

Supplement: S3 Fig — (A): Prediction of conserved active sites by InterPro. (B): The sequence alignment of CpMTA1 and its orthologs was performed using CLC Genomics Workbench. The red boxes represent the conserved sites of CpMTA1. (TIF) [file ppat.1012476.s004.tif]

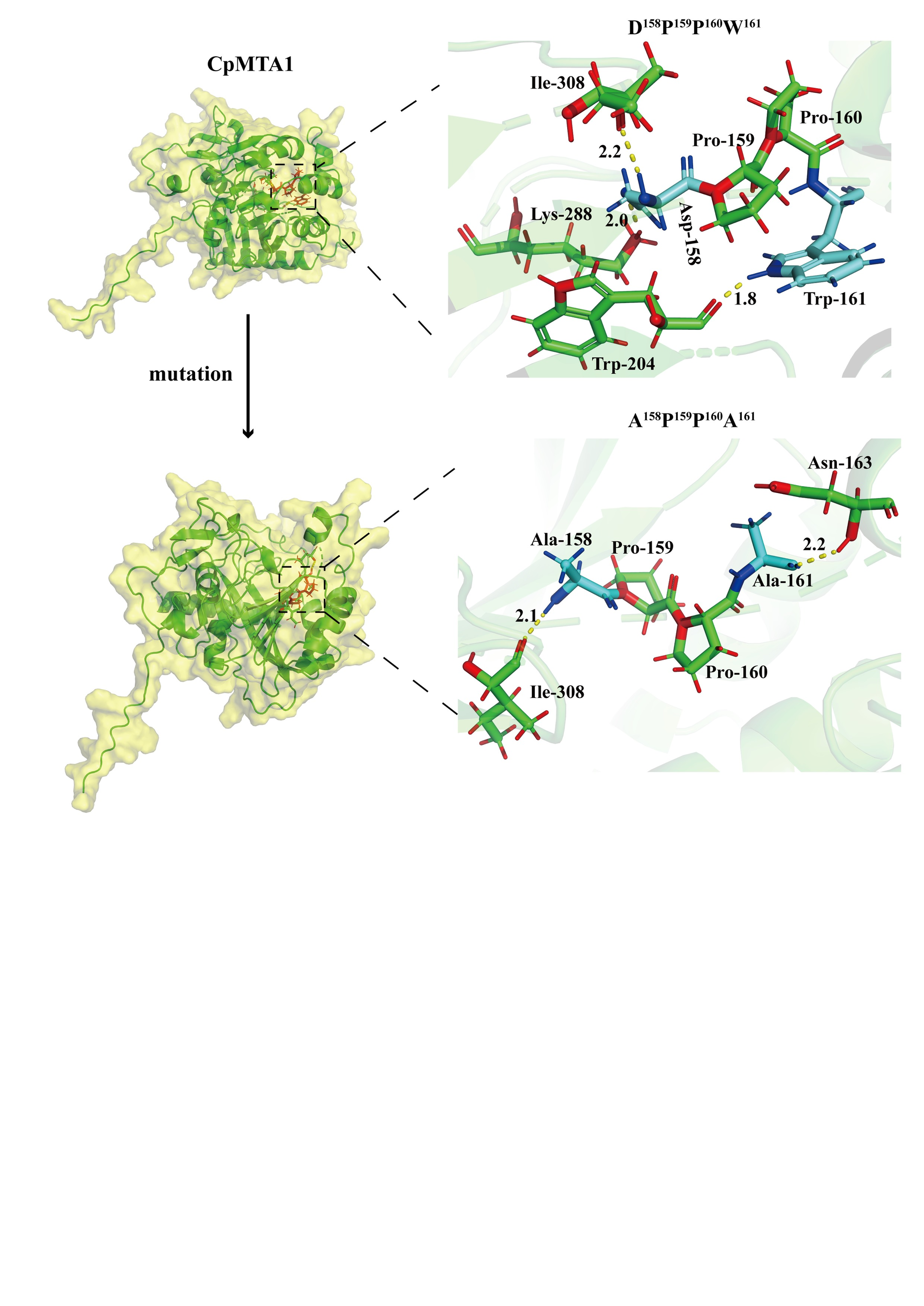

Supplement: S4 Fig — Asp-158, Pro-159, Pro-160, and Trp-161 are highlighted in red (left). To analyze the amino acid residues at positions 158 and 161 before and after mutation, PyMOL v2.5.4 was used to create a hydrogen bond interaction map. Amino acids 158 and 161 are shown in blue, and hydrogen bonds are represented by yellow dashed lines (right). (TIF) [file ppat.1012476.s005.tif]

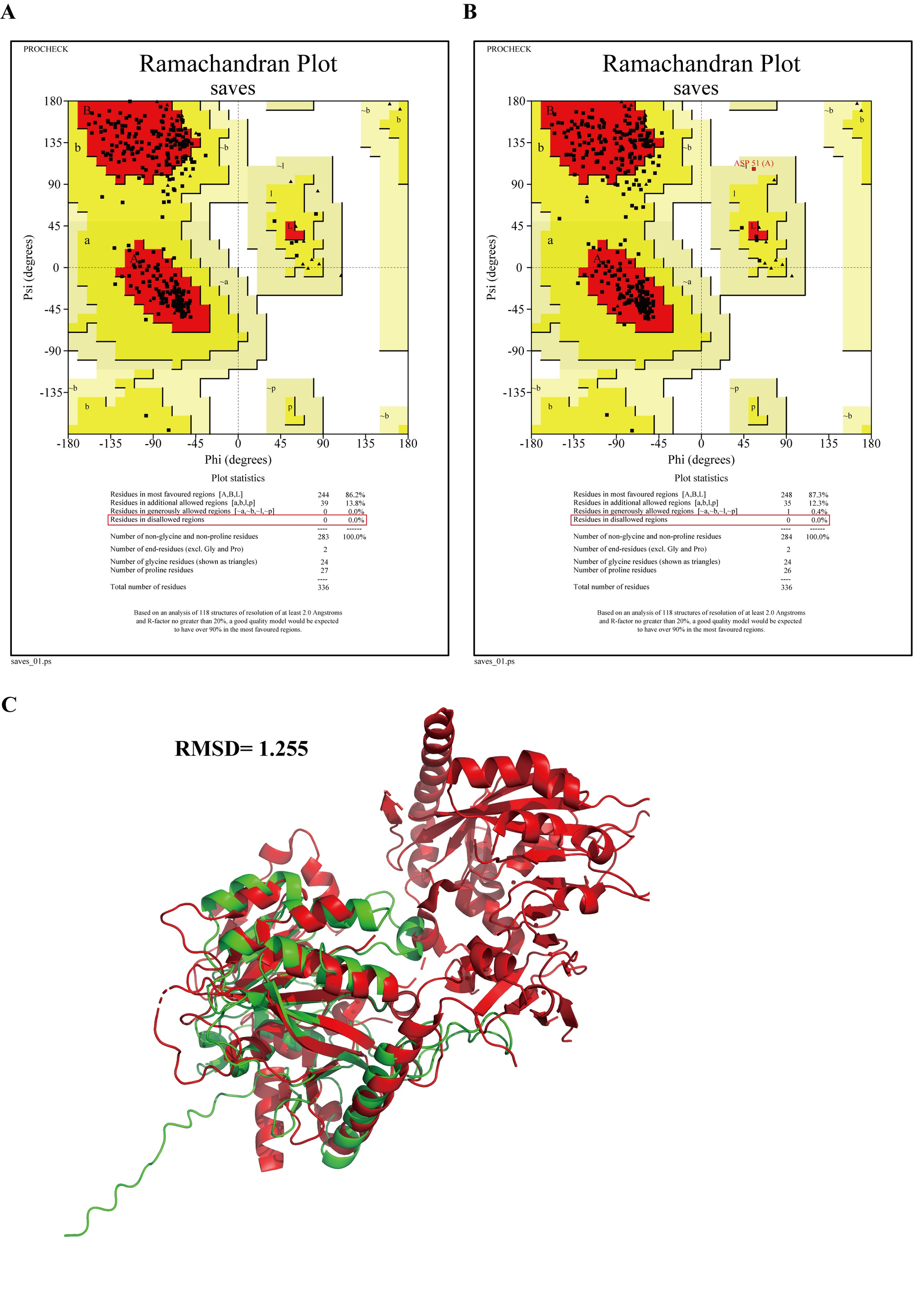

Supplement: S5 Fig — (A): Ramachandran plot of the predicted structure of wild-type CpMTA1 protein. (B): Ramachandran plot of the predicted structure of mutated CpMTA1 protein (APPA). (C): The predicted CpMTA1 structure by AlphaFold2 was compared with Arabidopsis METTL4 structure (PDB DOI: https://doi.org/10.2210/pdb7CVA/pdb) using PyMOL software. The root mean square deviation (RMSD) is 1.255, indicating that the similarity between the two proteins (Arabidopsis METTL4 is shown in red, CpMTA1 is shown in green). (TIF) [file ppat.1012476.s006.tif]

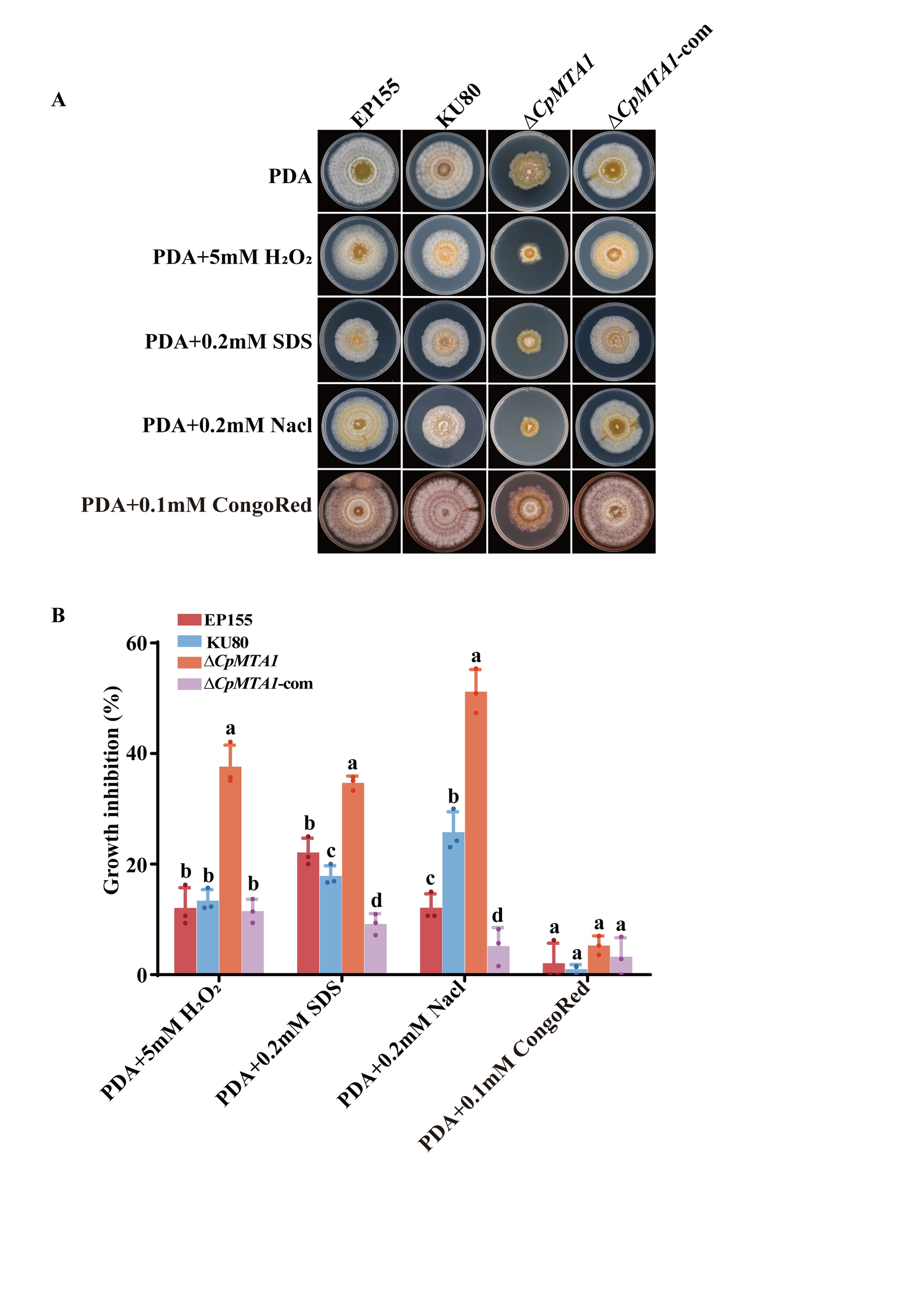

Supplement: S6 Fig — (A): Colonies of the wild type, deletion mutant, complementation mutant and overexpression mutant were shown after 7 days of cultivation at 26°C. (B): Growth inhibition rate of strains by stressors, and colony diameter on PDA was set to 100%. All measurements were performed after 7 days of growth at 26°C and were performed in triplicate. Error bars represent the standard deviation. Different letters on the bars indicate significant differences (p < 0.05). (TIF) [file ppat.1012476.s007.tif]

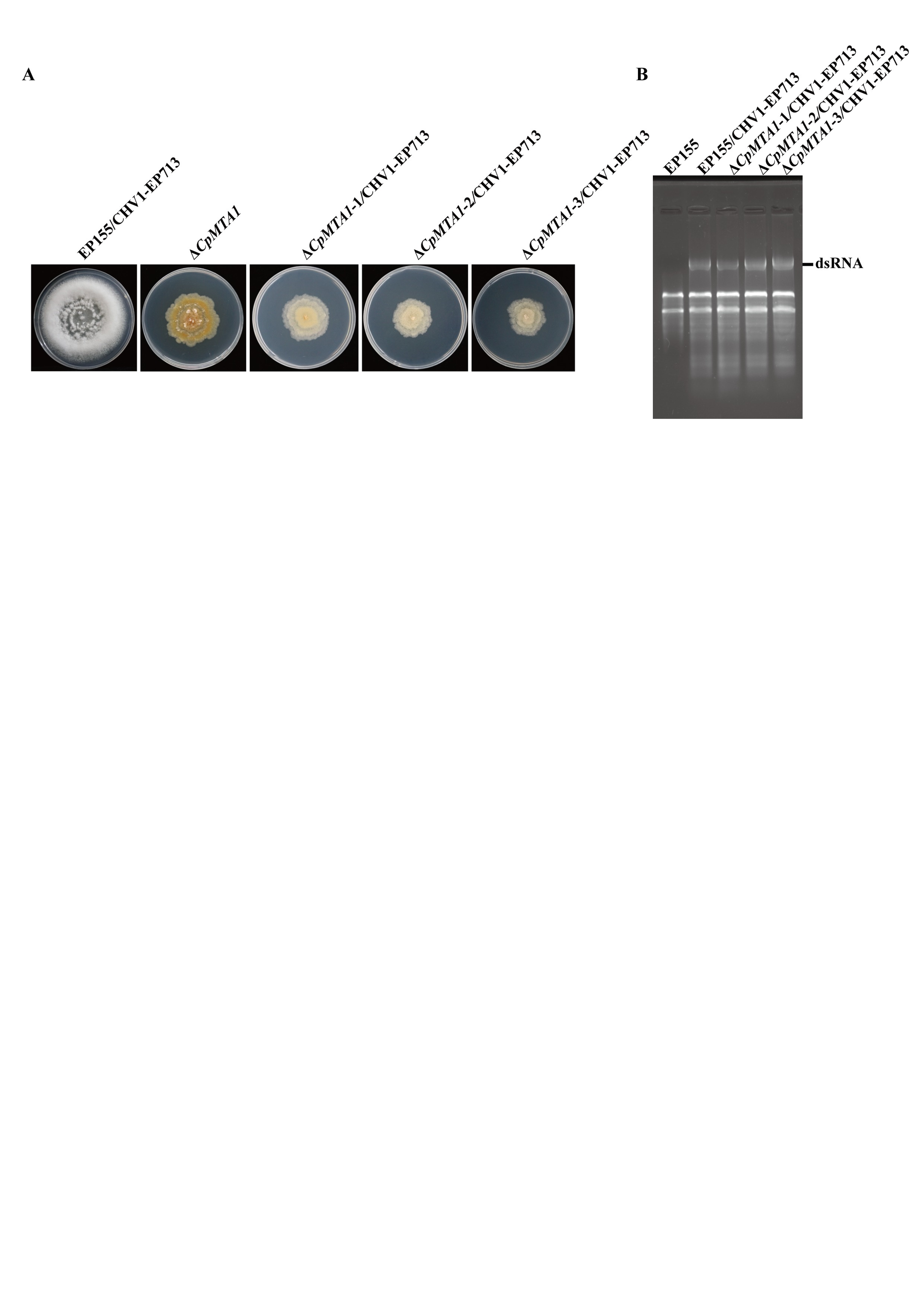

Supplement: S7 Fig — (A): The colony morphology of hypovirus-free and hypovirus containing ΔCpMTA1 mutants was observed on PDA at day 7 post-inoculation. (B): Viral dsRNA accumulation was analyzed using agarose gel electrophoresis in hypovirus-containing ΔCpMTA1 mutants. (TIF) [file ppat.1012476.s008.tif]

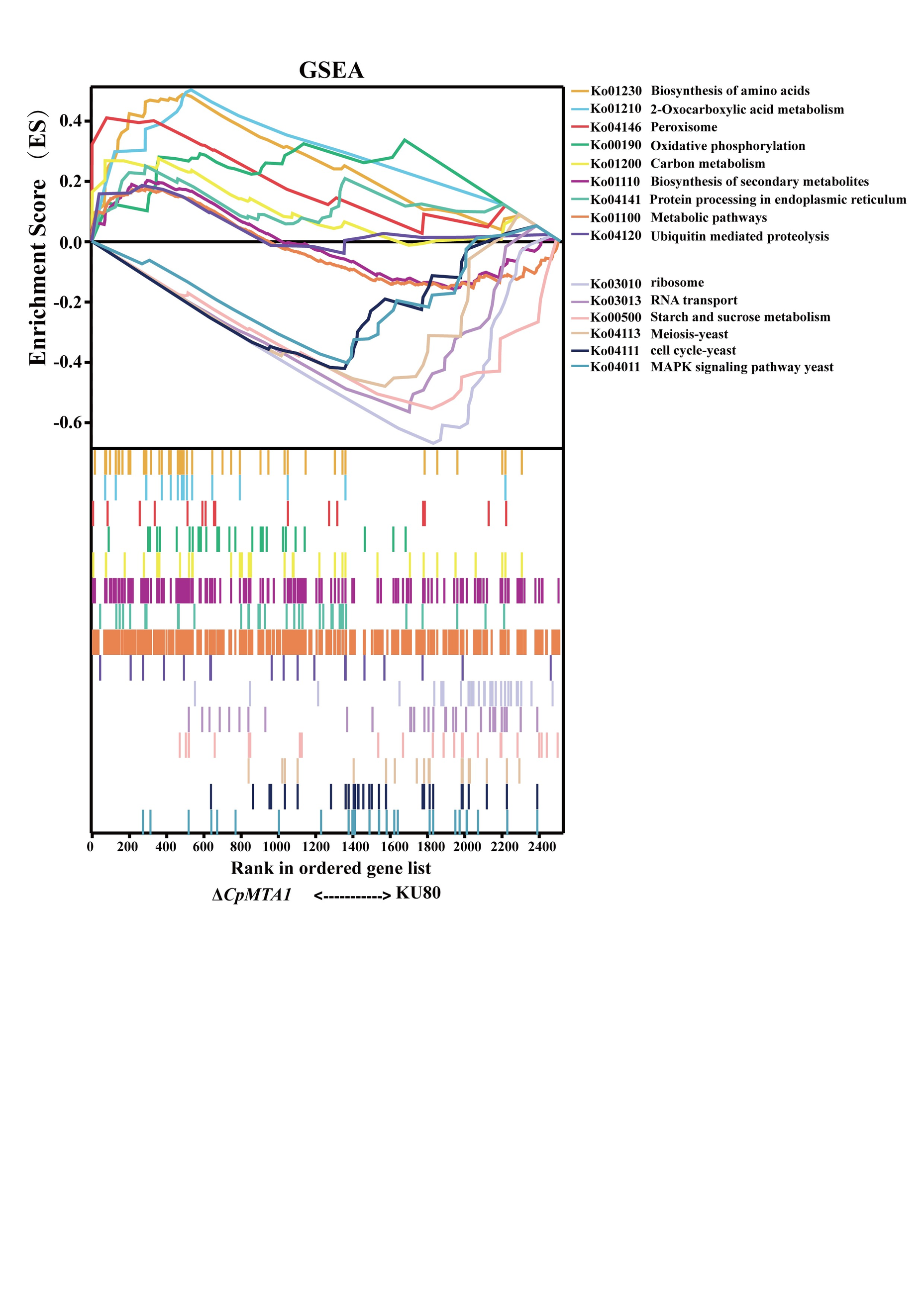

Supplement: S8 Fig — The top of the graph represents the enrichment score (ES): The ES value reflects the degree to which the members of the gene set are enriched at both ends of a sorted list. A positive ES indicates that the gene set is enriched at the top of the list, i.e., the pathway is activated or upregulated; while a negative ES indicates that the gene set is enriched at the bottom, i.e., the pathway is repressed or downregulated. The color of the curve is indicative of the color associated with the KEGG pathway listed on the right. The bottom of the graph shows the distribution of the rank values of all genes after sorting. (TIF) [file ppat.1012476.s009.tif]

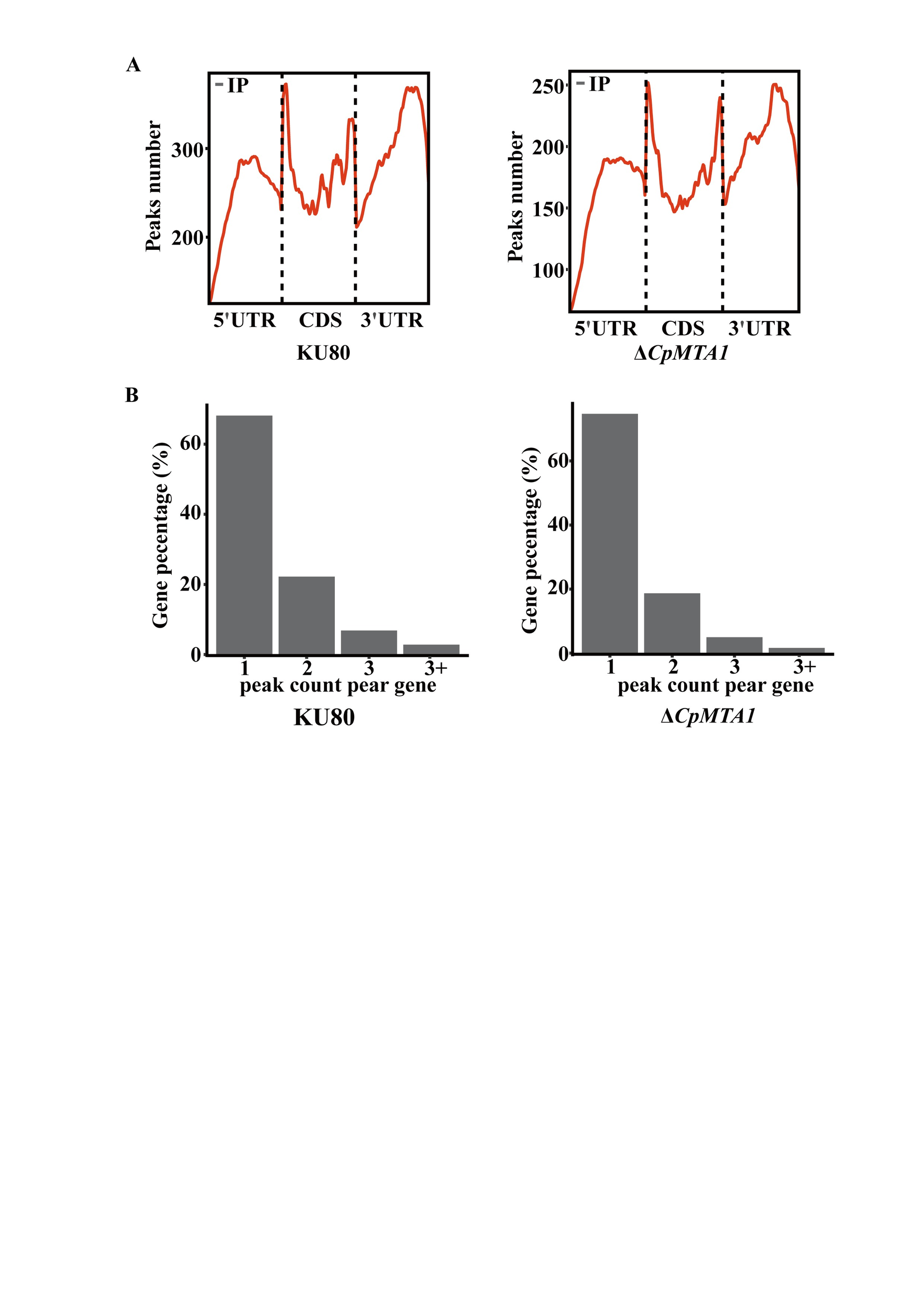

Supplement: S9 Fig — (A): Distribution of m6A peaks along the whole mRNA transcripts of C. parasitica detected in the KU80 strain and the ΔCpMTA1 mutant. (B): Number of the m6A-modified transcripts containing different m6A peak numbers in the KU80 strain and the ΔCpMTA1 mutant. (TIF) [file ppat.1012476.s010.tif]

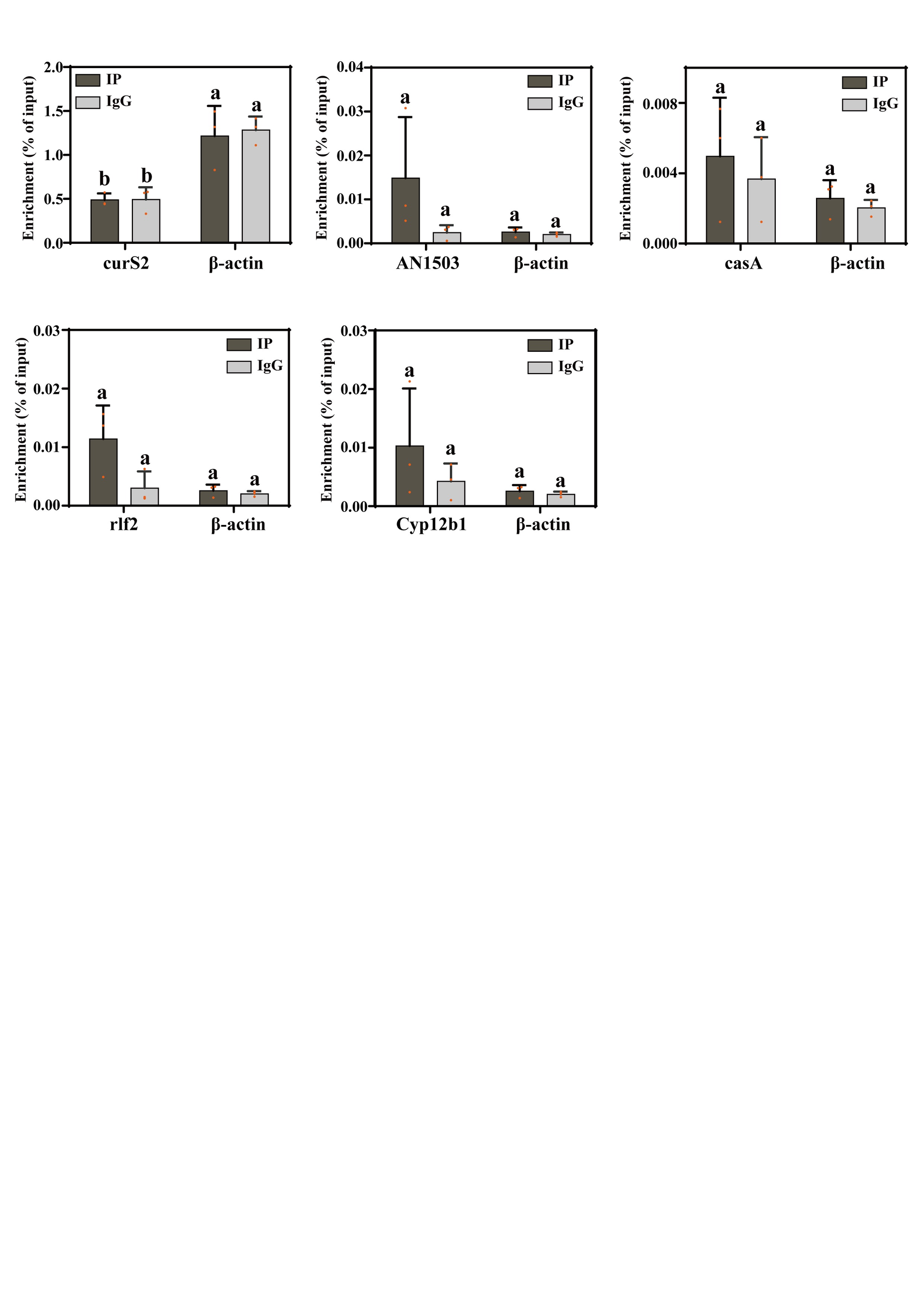

Supplement: S10 Fig — RIP assay was performed using an anti-flag antibody in EP155/3×flag-CpMTA1 strain. β-actin was used as a negative control. The fold enrichment values were normalized to that of Input. The same letters on the bars mean no significant difference between samples (ANOVA followed by Tukey’s test). (TIF) [file ppat.1012476.s011.tif]

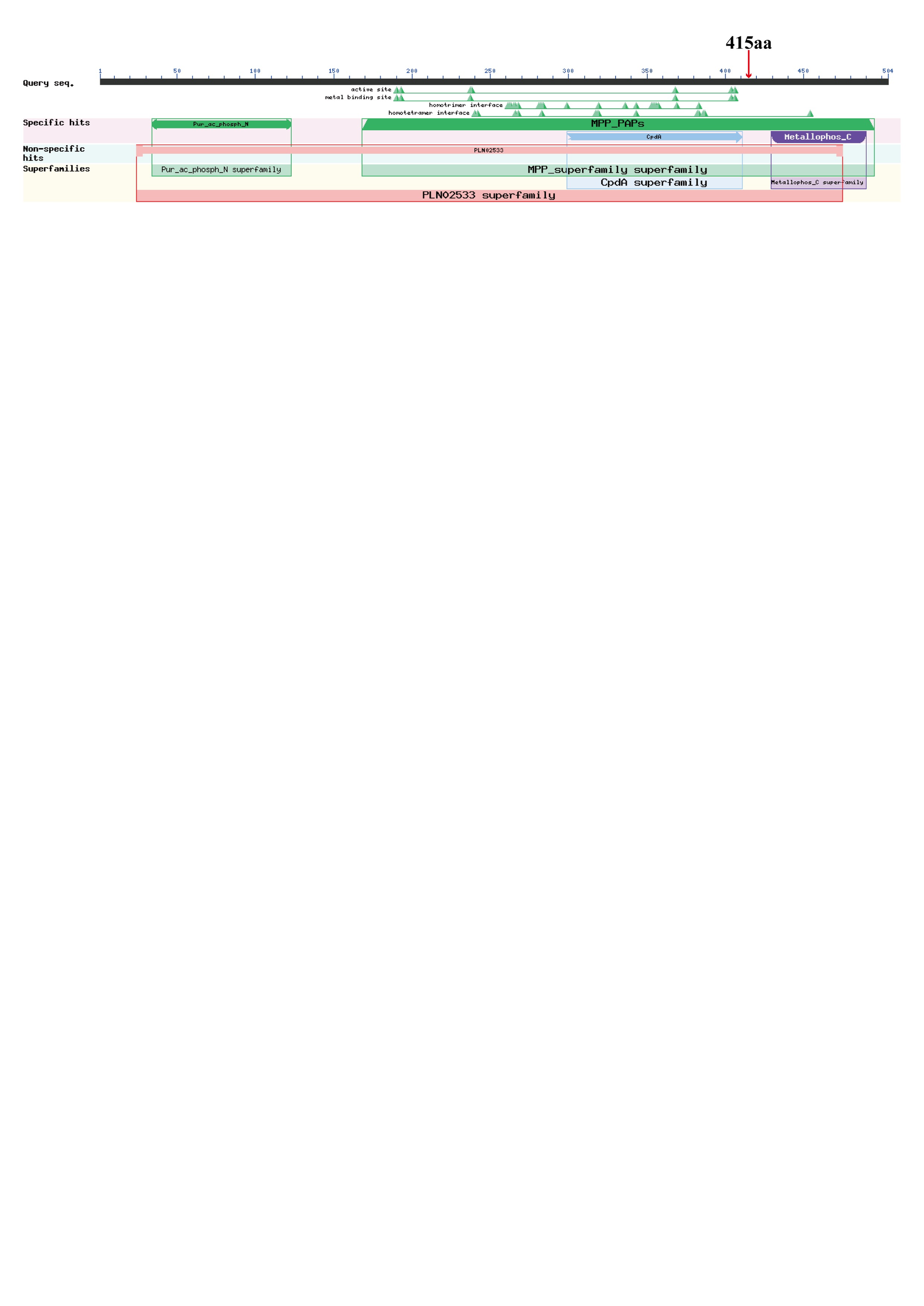

Supplement: S11 Fig — (TIF) [file ppat.1012476.s012.tif]

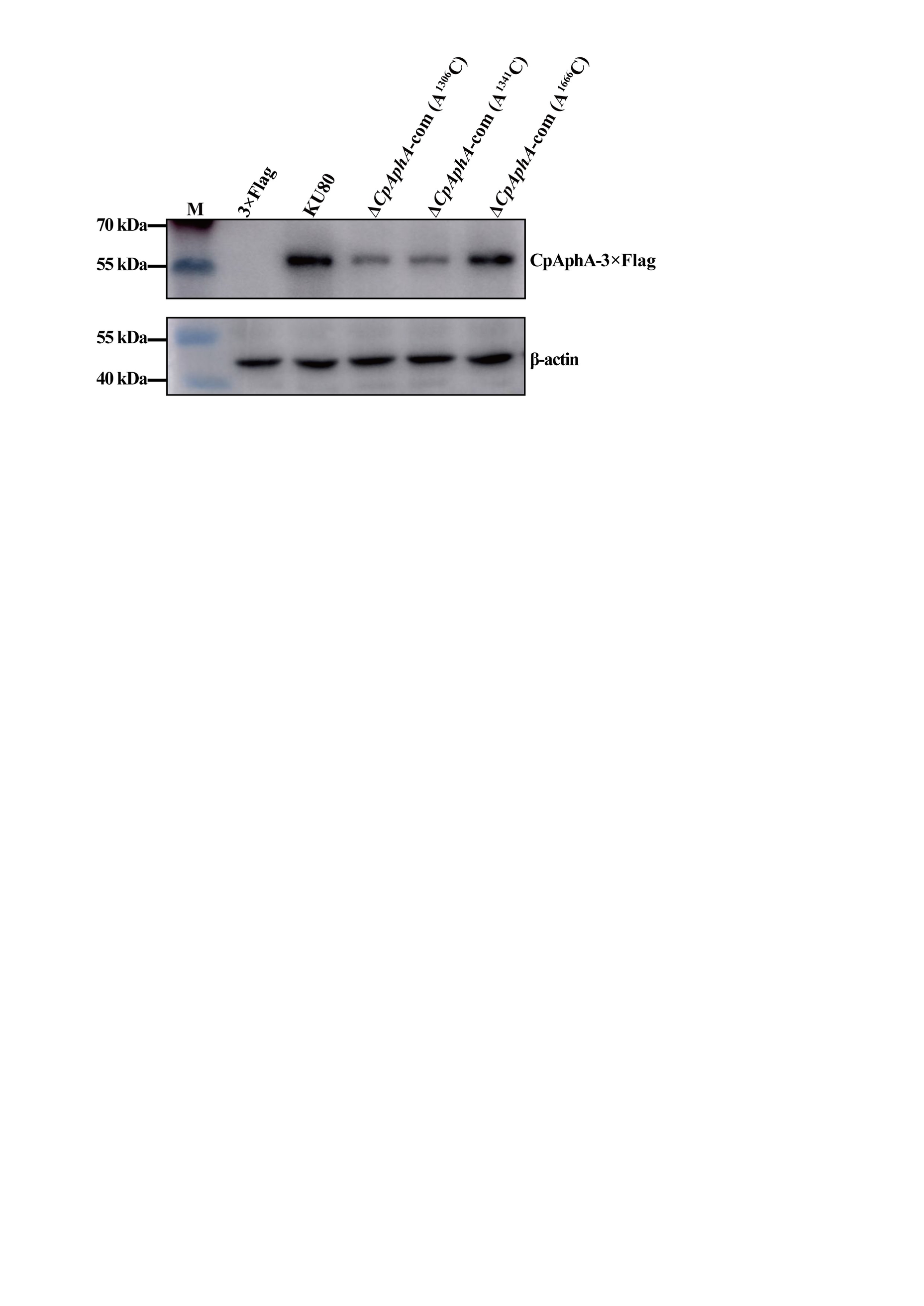

Supplement: S12 Fig — (TIF) [file ppat.1012476.s013.tif]

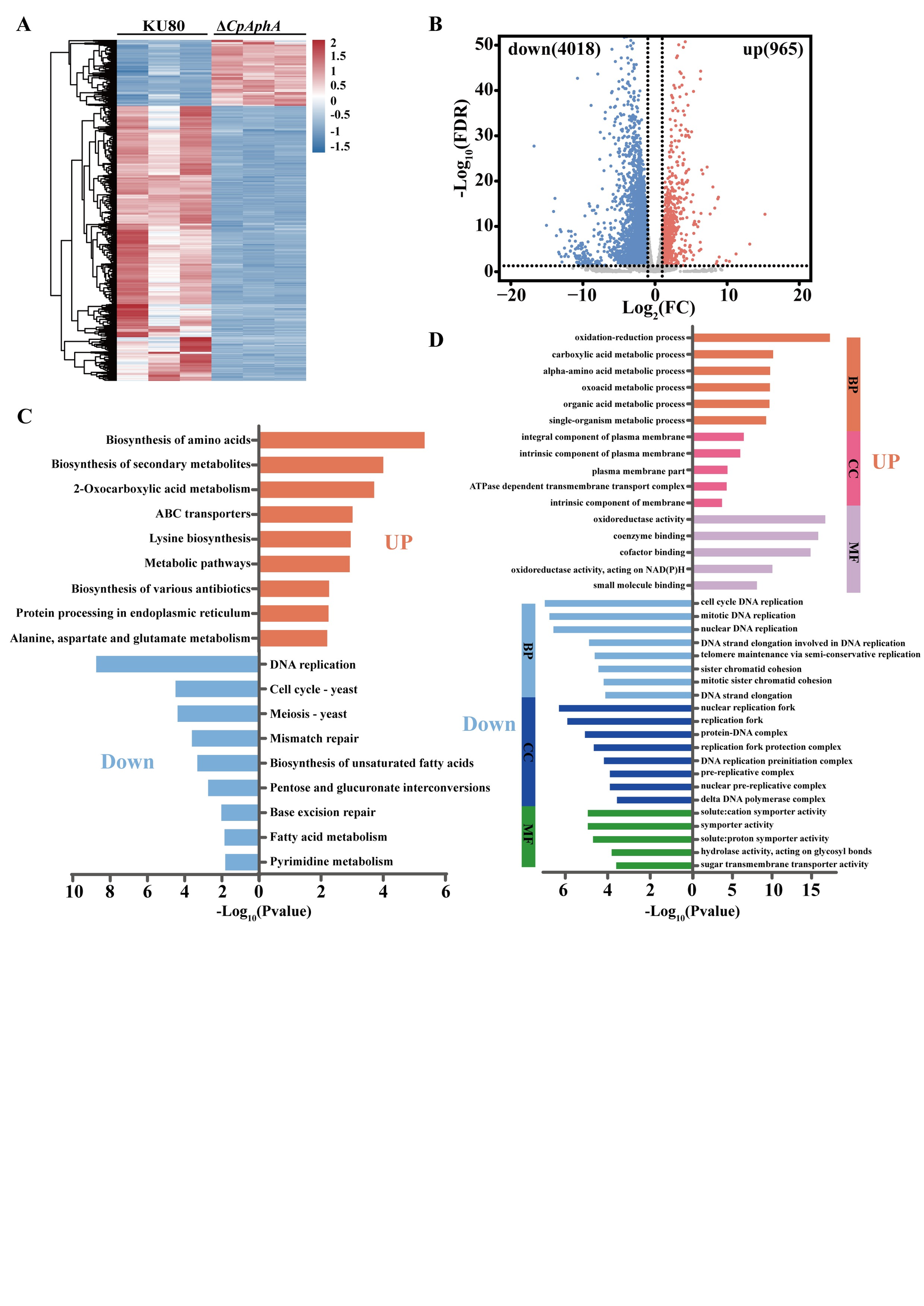

Supplement: S13 Fig — (A): Heatmap showing relative expression levels of genes in ΔCpAphA relative to KU80 strain based on the RNA-seq data. Cutoff criteria for differentially expressed genes included log2|FC|≥1 and FDR≤0.05 (red color indicates an increase and blue color represents a decrease in mRNA expression.) (B): Volcano plot showing the DEGs between KU80 and ΔCpAphA. (C): KEGG pathway enrichment analysis of DEGs. (D): GO-based enrichment analysis of DEGs in terms of biological process (BP), cell component (CC) and molecular function (MF). (TIF) [file ppat.1012476.s014.tif]
